# AI Algorithm for Lung Adenocarcinoma Pattern Quantification (PATQUANT): International Validation and Advanced Risk Stratification Superior to Conventional Grading

**DOI:** 10.1002/mco2.70380

**Published:** 2025-09-08

**Authors:** Yuan Wang, Kris Lami, Waleed Ahmad, Simon Schallenberg, Andrey Bychkov, Yuanzi Ye, Danny Jonigk, Xiaoya Zhu, Sofia Campelos, Anne Schultheis, Matthias Heldwein, Alexander Quaas, Ales Ryska, Andre L. Moreira, Junya Fukuoka, Reinhard Büttner, Yuri Tolkach

**Affiliations:** ^1^ Institute of Pathology University Hospital Cologne Cologne Germany; ^2^ Department of Pathology Informatics Nagasaki University Nagasaki Japan; ^3^ Institute of Pathology Charité Berlin Germany; ^4^ Department of Pathology Kameda Medical Center Kamogawa Japan; ^5^ Department of Pathology The First Affiliated Hospital of Anhui Medical University Hefei China; ^6^ Institute of Pathology University Hospital Aachen Aachen Germany; ^7^ German Center for Lung Research DZL BREATH Hanover Germany; ^8^ Department of Pathology of Run Run Shaw Hospital Affiliated to Zhejiang University Hangzhou China; ^9^ IMP Diagnostics Porto Portugal; ^10^ Institute of Pathology University Hospital Freiburg Freiburg Germany; ^11^ Department of Cardiothoracic Surgery University Hospital Cologne Cologne Germany; ^12^ The Fingerland Department of Pathology Charles University Medical Faculty Hospital Hradec Králové Czech Republic; ^13^ Department of Pathology NYU Grossman School of Medicine New York New York USA

**Keywords:** AI, grading, lung adenocarcinoma, lung cancer, pattern, PATQUANT

## Abstract

The morphological patterns of lung adenocarcinoma (LUAD) are recognized for their prognostic significance, with ongoing debate regarding the optimal grading strategy. This study aimed to develop a clinical‐grade, fully quantitative, and automated tool for pattern classification/quantification (PATQUANT), to evaluate existing grading strategies, and determine the optimal grading system. PATQUANT was trained on a high‐quality dataset, manually annotated by expert pathologists. Several independent test datasets and 13 expert pathologists were involved in validation. Five large, multinational cohorts of resectable LUAD (patient *n* = 1120) were analyzed concerning prognostic value. PATQUANT demonstrated excellent pattern segmentation/classification accuracy and outperformed 8 out of 13 pathologists. The prognostic study revealed a distinct prognostic profile for the complex glandular pattern. While all contemporary grading systems had prognostic value, the predominant pattern‐based and simplified IASLC systems were superior. We propose and validate two new, fully explainable grading principles, providing fine‐grained, statistically independent patient risk stratification. We developed a fully automated, robust AI tool for pattern analysis/quantification that surpasses the performance of experienced pathologists. Additionally, we demonstrate the excellent prognostic capabilities of two new grading approaches that outperform traditional grading methods. We make our extensive agreement dataset publicly available to advance the developments in the field.

## Graphical abstract

• PATQUANT is a clinical‐grade designed for fully automated, quantitative analysis of whole‐slide images from lung adenocarcinoma (LUAD) patients.

• In a large international study assessing interobserver agreement, PATQUANT outperformed 8 out of 13 expert pathologists.

• Conventional LUAD grading systems, integrated within PATQUANT, enable robust risk stratification.

• Two new, explainable grading systems have been proposed and validated in a large patient cohort.

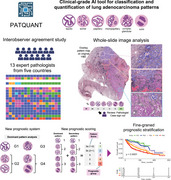

## 1 Introduction

Lung adenocarcinoma (LUAD) is the most common subtype of non‐small cell lung cancer [1]. Pathological assessment and histological grading of adenocarcinoma provide important information about tumor aggressiveness and prognosis [2]. Several histological grading systems are in use that are based on the evaluation of tumor architecture and detection/quantification of distinct patterns [2, 3]. The WHO classification suggests five morphological patterns (lepidic, acinar, papillary, micropapillary, and solid) with a three‐tier grading based on the dominant pattern (G1–lepidic, G2–acinar and papillary, G3–micropapillary and solid) [2]. The grading system proposed by the International Association for Study of Lung Cancer (IASLC), also recognized by the WHO, defines one additional pattern (complex glandular: cribriform and fused glands) as a high‐risk pattern based on the available evidence [4, 5, 6] and classifies G1 and G2 tumor with > 20% of high‐risk patterns (micropapillary, complex glandular, solid) as G3 [3]. Both systems, compared directly, provide meaningful stratification of patients with resectable LUAD according to their risk of survival and progression [3].

Digitization and application of computational pathology methods to pathology image analysis allow for the development of deep learning (DL)‐based tools for automated image analysis [7, 8]. Several of such tools, including those from our group, have shown clinical‐grade performance on par with expert pathologists in various tumors [9, 10, 11, 12, 13] including lung cancer [14]. Although several artificial intelligence (AI) tools have been developed for pattern recognition in LUAD, most of them have substantial limitations, such as limited training data, the absence of adequate validation for interobserver agreement and prognostic role, non‐recognition of the complex glandular pattern as a separate pattern, or not directly comparing different grading systems [15, 16, 17, 18, 19, 20, 21, 22].

**FIGURE 1:**
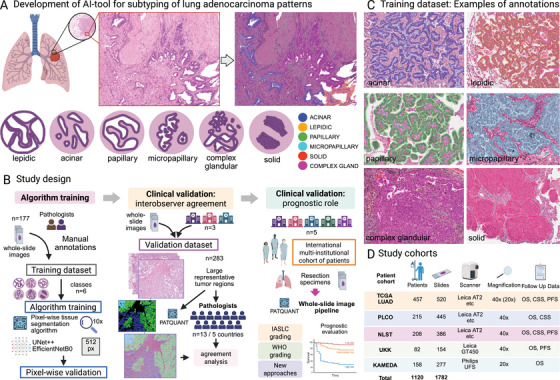
PATQUANT principle, study design, and patient cohorts. (A) PATQUANT is a deep learning algorithm that analyzes hematoxylin & eosin‐stained images and performs pixel‐wise segmentation of the six lung adenocarcinoma (LUAD) patterns. (B) Study design outlining principles of algorithm training and clinical validation concerning interobserver agreement and prognostic value. (C) Principles and examples of manual annotations (examples of manual pattern annotations from QuPath software). (D) Study cohorts used for prognostic analysis. Created in BioRender. Tolkach, I. (2025) https://BioRender.com/q10s060.

In this study, we developed PATQUANT, a powerful pixel‐wise AI‐based Pattern Quantification and Analysis Tool (PATQUANT) based on a large, high‐quality training dataset manually annotated by expert pathologists and implemented as a fully automated whole‐slide image (WSI) analysis pipeline providing quantitative and visual outputs for pathologists (Figure 1A–C). We performed a comprehensive interobserver agreement study involving a large dataset and 13 pathologists from five countries. Using five different multi‐national, well‐characterized cohorts of patients with resectable LUAD (Figure 1D), we carried out the validation of PATQUANT's prognostic value and suggested and implemented two new approaches for histological grading. These allow for fine‐granular prognostic stratification of patients superior to that using WHO/IASLC systems. We fully release our large interobserver agreement dataset with pathologists’ scores to advance education and further research efforts in this domain.

## 2 Results

### 2.1 Development of the Algorithm

The key component of the developed PATQUANT for LUAD is a pixel‐level segmentation algorithm trained on a large, high‐quality dataset that was manually annotated by experts (Figure 1A,C). Six distinct patterns were employed according to the IASLC proposal [3]. The model analyses WSIs at 10x magnification. The parameters of the final version of the model are outlined in Figure 1B (for details on development, see Methods). Next, we validated PATQUANT at the pixel‐wise level (segmentation/classification accuracy), in an interobserver agreement study (with 13 pathologists from 5 countries), and for prognostic value using five well‐characterized cohorts of patients with resectable LUAD (Figure 1B). Moreover, we propose, implement, and validate two new pattern‐based grading principles.

### 2.2 Formal Validation and Detailed Analysis of Pattern Classification Overlaps

**FIGURE 2:**
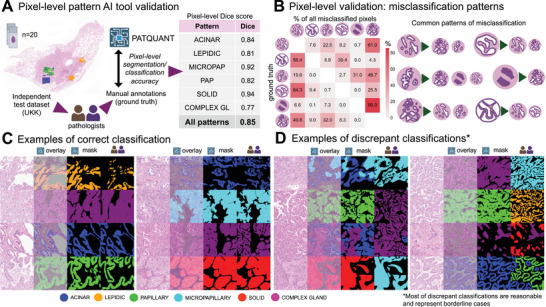
Formal validation of pattern segmentation/classification accuracy and analysis of discrepant cases. (A) PATQUANT was validated using an independent test dataset (WSI *n* = 20) manually annotated by pathologists (the cases and annotations are shown in Figure S1). The Dice score (or F1 score) measures how well the ground truth (manual annotations) and AI tool predictions overlap (range: 0–1). The average Dice score of 0.85 corresponds to excellent segmentation precision. (B) Analysis of discrepant segmentations/classifications. The confusion matrix on the left side shows the structure of “misclassified” pixels (=area) for single patterns (horizontally); percentages from all discrepant pixels classified as other patterns are presented. Right plate summarizes typical patterns of misclassifications. As expected, these misclassifications occurred in areas of typical morphological pattern overlap and are consistent with the typical difficulties experienced by pathologists. (C) Examples of correct classification showing excellent performance of PATQUANT for pattern recognition. Shown for each region from left to right are the original H&E image, overlay of algorithm prediction, segmentation mask of algorithm, and ground truth (manual annotation), respectively. (D) Examples of discrepant classifications showing that most of these are cases with overlapping morphologies for several patterns. Created in BioRender. Tolkach, I. (2025) https://BioRender.com/q10s060.

For formal validation, we use a high‐quality, manually annotated independent test dataset (UKK; see Figure S1 and Methods) representative of all patterns. In a comparative analysis of manual annotations and PATQUANT predictions for six patterns, the average Dice score (F1 score) for segmentation/classification accuracy for all patterns was 0.85, indicating excellent pixel‐wise accuracy (Figure 2A). Since patterns are known to exhibit significant morphological overlap, we analyzed and quantified the areas (as % of all misclassified pixels) where the model's predictions deviated from the annotation ground truth (Figure 2B). As expected, these misclassifications occurred in areas of typical morphological pattern overlap, for example, acinar pattern detected as a complex glandular pattern and vice versa, or acinar pattern detected as papillary and vice versa. All pattern misclassifications are summarized in Figure 2B and are consistent with the well‐known difficulties experienced by pathologists when dealing with morphological overlaps [22]. A detailed visual evaluation confirmed PATQUANT's excellent performance in most cases (Figure 2C), while borderline classifications accounted for the most discrepant cases (Figure 2D).

### 2.3 Interobserver Agreement Analysis

**FIGURE 3:**
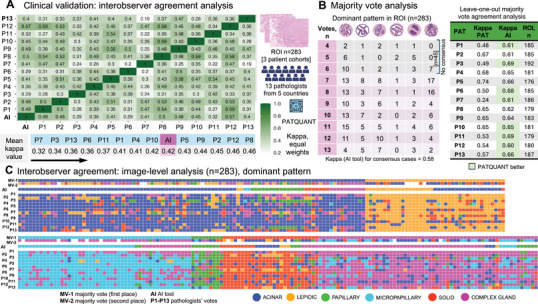
Interobserver agreement study. (A) Confusion matrix showing agreement analysis between single graders including PATQUANT. A large dataset of regions of interest (ROIs) was created using whole‐slide images (WSIs) of patients from three cohorts representing three different countries (the United States, Germany, and Japan), different laboratory and digitization practices (three different histoscanners). The metric used is the kappa statistics with equal weights. The average kappa values are shown for each grader showing that PATQUANT outperformed 8 out of 13 participating pathologists. (B) Majority vote analysis for dominant pattern. On the left side, the structure of ROIs according to pattern and majority vote decision is shown subdivided into the cases where pathologists reached consensus (> 50% pathologists providing the same dominant pattern) or not. Leave‐one‐out majority vote agreement analysis is shown on the right side, where a single pathologist is compared to the majority vote of the other graders, excluding that pathologist and PATQUANT. PATQUANT was excluded from all majority vote quantifications and outperformed 8 out of 13 pathologists. (C) Heatmap showing the dominant pattern provided for all 283 ROIs by pathologists and PATQUANT. The two upper rows represent the major voting (only pathologists) results: majority vote (MV‐1) and the second most frequent pattern according to pathologists’ scores (MV‐2). Comment: Simple agreement‐based analysis is provided in Figure S2. Created in BioRender. Tolkach, I. (2025) https://BioRender.com/q10s060.

We created a large, representative dataset of 283 tumor regions (ROI) from WSIs of three patient cohorts (Germany/Europe, Japan, the United States), representing heterogeneous populations known for molecular genetic diversity, tumor morphologies, and laboratory and digitization practices (utilizing three different histoscanners; see details in Methods). These ROIs were evaluated by 13 pathologists specializing in lung cancer from five different countries and by PATQUANT. A detailed agreement analysis for dominant pattern identification is presented in Figure 3A–C. PATQUANT demonstrated significant agreement with pathologists, outperforming 8 out of 13 pathologists when average agreement (equal‐weight kappa metric) was analyzed from comprehensive single‐grader agreement comparisons (Figure 3A; for simple agreement metric see Figure S2).

In general, the interobserver agreement between pathologists and the AI tool was low to moderate (kappa range for pathologists: 0.32–0.46; AI tool: 0.42) reflecting the complexity of pattern subtyping due to significant morphological overlaps among patterns. We then performed a majority vote analysis. For 45 ROIs, a consensus among pathologists could not be reached, while for the remaining 238 ROIs, at least 50% of pathologists identified the same dominant pattern (Figure 3B). The kappa score for consensus cases for PATQUANT was significantly higher (0.58 vs. 0.42 for all cases), implying that consensus cases were more straightforward.

**FIGURE 4:**
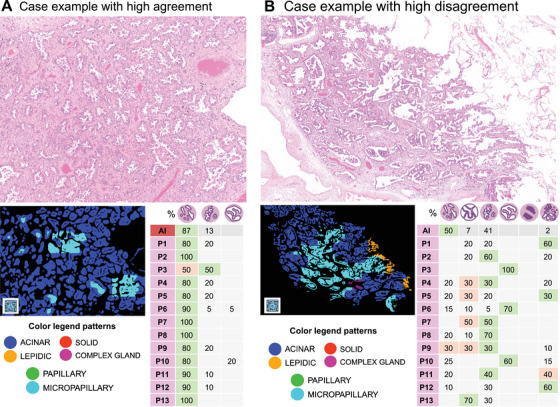
Examples of the ROIs from the interobserver agreement study. (A) An example of ROI with high agreement levels among pathologists. The detailed structure of detected patterns is provided for PATQUANT and pathologists. (B) An example of ROI with low agreement levels among pathologists. The detailed structure of detected patterns is provided for PATQUANT and pathologists. Created in BioRender. Tolkach, I. (2025) https://BioRender.com/q10s060.

In the leave‐one‐out majority vote agreement analysis (where a single pathologist is compared to the majority vote of the other experts, excluding that pathologist and PATQUANT; PATQUANT was excluded from all majority vote quantifications), the AI tool outperformed 8 out of 13 pathologists (Figure 3B). A detailed ROI‐level analysis (Figure 3C) showed that the AI tool's deviations from the ground truth, as established by majority voting, are most evident in cases where pathologists’ agreement on the dominant pattern is also low. In such cases, pathologists sometimes provided up to 4 different opinions on the dominant pattern (Figure 3C). Representative examples of cases with high and low agreement are shown in Figure 4A,B.

Moreover, for three experienced pathologists (P6, P12, and P13), we compared how AI assistance effects the interobserver agreement. After wash‐out period of 9 months, the pathologists re‐evaluated all 283 images, now seeing visual outputs (overlays) and % breakdown for patterns generated by PATQUANT. The interobserver agreement in case of AI‐assistance was significantly higher compared to the original evaluation (initial 0.37‐0.40, with AI‐assistance 0.67‐0.78; details in Figure S3) demonstrating significant impact of PATQUANT for decreasing subjectivity of assessment.

### 2.4 WSI Analysis Pipeline

**FIGURE 5:**
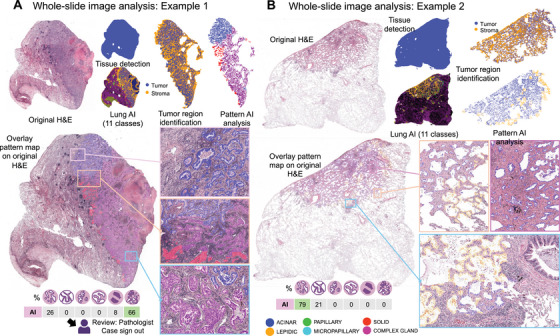
WSI analysis pipeline. Whole slide image pipeline consists of tissue detection module, multi‐class tissue segmentation algorithm for lung cancer developed earlier, and PATQUANT. Multi‐class tissue segmentation algorithms detects 11 different classes including tumor and all tumor‐associated classes, for example, tumor stroma, necrosis, and so forth (color legend: tumor—blue, stroma—orange, other classes are shown in various other classes). In regions detected as epithelial tumor tissue, PATQUANT performs the pattern analysis resulting in the segmentation mask with colors corresponding to six distinct patterns (color legend is provided at the bottom part of the figure). PATQUANT provides quantitative metrics in absolute area and in percentages of different patterns at slide level and aggregates the information from multiple slides of the same patient, when available. It also provides high‐resolution overlays of segmentation masks on the original images facilitating review by pathologists. Two examples are shown in Figures (A) and (B). High‐resolution versions of these images are provided in Figures S3–S6 , with further examples in Figure S7–S15. Created in BioRender. Tolkach, I. (2025) https://BioRender.com/q10s060.

The PATQUANT was implemented as a WSI analysis pipeline that enables fully automated processing of WSIs and aggregates information from multiple WSIs of a single case (Figure 5). This pipeline includes tissue detection (and quality control) as pre‐analytical steps, as well as two segmentation models: a previously developed multi‐class analysis tool for lung cancer to detect tumor regions [14] and PATQUANT to analyze and quantify patterns in the tumor area. The pipeline provides fully quantitative outputs at both the slide and case levels and generates high‐resolution overlay masks of the patterns for visual analysis by pathologist at any magnification. Representative examples of WSI analysis are shown in Figure 5A,B (higher resolution in Figures S4–S7 ) and in Figures S8–S16.

### 2.5 Prognostic Role of Patterns and Pattern‐Based Grading

**FIGURE 6:**
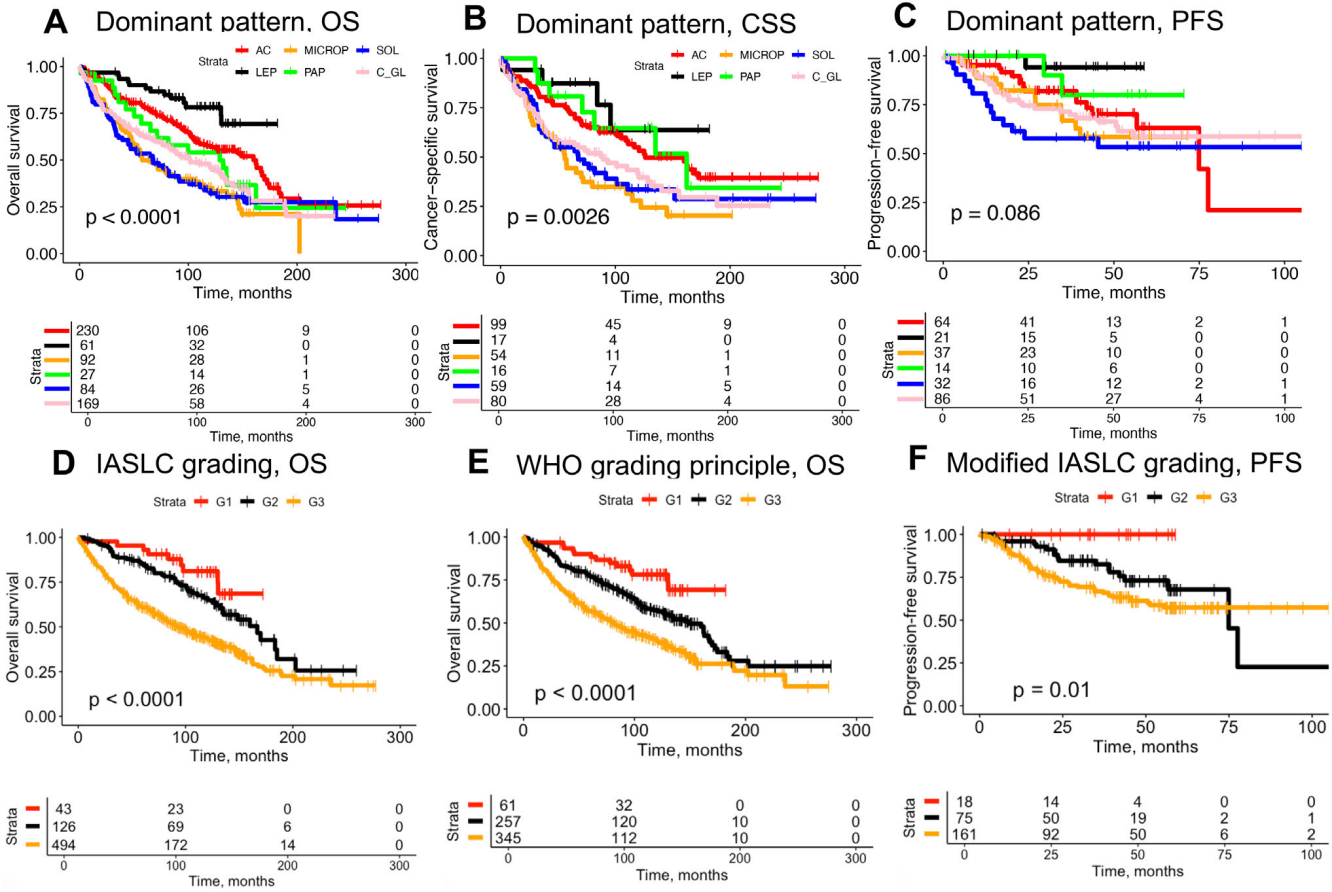
Prognostic study: single patterns and contemporary grading systems. (A) Kaplan–Meier estimates with log‐rank test. Cohort: merged cohort (PLCO, NLST, UKK, KAMEDA). TCGA cohort was excluded from these analyses as selection bias was identified for lepidic‐predominant tumors (for details, see Results, Figures S16–S18). Endpoint: overall survival (OS), stratification based on a dominant pattern. Notably, a distinct prognostic profile can be seen for complex glandular pattern, indicating moderate risk of negative outcome, compared to classical very low/low‐grade patterns (lepidic; acinar, papillary) and high‐grade patterns (micropapillary, solid). (B) Kaplan–Meier estimates with log‐rank test. Cohort: merged cohort (PLCO, NLST, UKK, KAMEDA). Endpoint: cancer‐specific survival (CSS), stratification based on a dominant pattern. (C) Kaplan–Meier estimates with log‐rank test. Cohort: merged cohort (PLCO, NLST, UKK, KAMEDA). Endpoint: progression‐free survival (PFS), stratification based on a dominant pattern. (A–C). Results of univariate Cox analysis are provided in Table S1. (D) Grading according to IASLC‐proposed grading system considering re‐classification of G1 and G2 cases with > 20% high‐risk patterns (micropapillary, complex glandular, solid) as G3. Kaplan–Meier estimates with log‐rank test. Cohort: merged cohort (PLCO, NLST, UKK, KAMEDA). Endpoint: OS. Univariate/multivariate Cox analysis results in Table S2. *Comment*: Detailed investigation of high‐risk percentage thresholds in Figure S20–S22. (E) Grading according to WHO‐recommended grading system, considering only dominant pattern (G1–lepidic, G2–acinar, papillary, G3–solid, micropapillary, complex glandular). Kaplan–Meier estimates with log‐rank test. Cohort: merged cohort (PLCO, NLST, UKK, KAMEDA). Endpoint: OS. Univariate/multivariate Cox analysis results in Table S3. *Comment*: This system outperforms the IASLC system (D) and shows independent prognostic value in multivariate Cox analysis incorporating common clinicopathological variables (pT, pN, age). Additionally, Figure S19 shows the simplified IASLC system proposed by Bossé et al. [23], which is also superior to the native IASLC system (Table S4 for uni/multivariate Cox analysis showing independent value). (F) Modified IASLC system (50% of high‐grade patterns in sum is allowed, none of them dominant), for details of systematic tests, see Figure S20–S22. Kaplan–Meier estimates with log‐rank test. Cohort: merged cohort (PLCO, NLST, UKK, KAMEDA). Endpoint: PFS.

To evaluate the prognostic value of PATQUANT, we conducted a large‐scale validation using five well‐characterized patient cohorts with resectable LUAD (Figure 1D, detailed clinicopathological characteristics provided in Table 1) originating from three countries (Germany, Japan, the United States) and representing different patient populations (also from molecular genetic point of view), tumor stages, morphologies, laboratory and digitization practices (using three different histoscanners, Figure 1D). All analyses were aggregated from multiple slides of single patients, when available. First, we assessed the prognostic value of single patterns as dominant patterns in individual cases for overall survival (OS), cancer‐specific survival (CSS), and progression‐free survival (PFS) endpoints (Figure 6A–C ; OS data were available in all cohorts allowing for greater statistical power, while CSS and PFS data were available only in some cohorts; univariate Cox analysis in Table S1). This analysis provided evidence for risk stratification, aligned with the classical concept of very low (lepidic), low (acinar and papillary), and high‐grade (micropapillary, solid, and complex glandular) patterns. Several important observations were made. First, tumors with identified dominant lepidic pattern in the TCGA cohort showed survival trends similar to high‐grade patterns (analysis for TCGA and merged cohort with and without TCGA in Figures S17–S19). The TCGA cohort has known biases, one of which is the selection of tumor regions with higher tumor content for sequencing and a patient population with more aggressive tumors. Moreover, the TCGA cohort was the only cohort in which, for most patients, only one slide was available (Table 1, multiple slides in 4.9% of cases, compared to 77.2%, 72.6%, 61.0%, and 39.9% of cases in PLCO, NLST, UKK, and KAMEDA cohorts, respectively), precluding robust case‐level estimation. We explain this prognostic behavior of lepidic‐predominant tumors in TCGA cohort as a consequence of potential high‐grade component in other slides from the same patients. In the merged dataset of the four other cohorts, lepidic predominant tumors had a clearly distinguishable favorable prognostic course (Figure 6A–C). Second, complex glandular pattern‐predominant cases showed more favorable outcomes (Figure 6A–C), implying an intermediate risk profile that we describe in detail later.

**TABLE 1:** Clinicopathological characteristics of study cohorts.

Parameter	TCGA	PLCO	NLST	UKK	KAMEDA
Patients, *n*	457	215	208	82	158
Whole slide images, *n*	520	445	386	154	277
Whole slide image *n* > 1, cases	21 (4.6%)	166 (77.2%)	151 (72.6%)	50 (61.0%)	63 (39.9%)
Whole slide image *n*, range	1–10	1–3	1–4	1–5	1–8
Sex					
Female	247 (54.1%)	112 (52.1%)	102 (49%)	41 (50.0%)	76 (48.1%)
Male	219 (45.9%)	103 (47.9%)	106 (51%)	41 (50.0%)	82 (51.9%)
Age					
Minimum	33	52	55	44	42
Maximum	88	74	74	87	85
Mean	64.9	63.6	64.0	65.2	66.9
Median	65.0	64.0	64.0	66.0	67.5
pT stage					
pT1	157 (34.4%)	111 (51.6%)	148 (71.2%)	41 (50.0%)	120 (75.9%)
pT2	240 (52.5%)	86 (40.0%)	39 (18.8%)	17 (20.7%)	27 (17.1%)
pT3	41 (9.0%)	5 (2.3%)	13 (6.3%)	12 (14.6%)	6 (3.8%)
pT4	16 (3.5%)	13 (6.0%)	6 (2.9%)	12 (14.6%)	3 (1.9%)
Unavailable	3 (0.7%)	−	2 (1.0%)	−	2 (1.3%)
pN stage					
pN0	300 (65.6%)	169 (78.6%)	151 (72.6%)	50 (61.0%)	133 (84.2%)
pN1	89 (19.5%)	23 (10.7%)	24 (11.5%)	14 (17.1%)	15 (9.5%)
pN2‐pN3	57 (12.5%)	22 (10.2%)	18 (8.7%)	18 (22.0%)	10 (6.3%)
Unavailable	11 (2.4%)	1 (0.5%)	15 (7.2%)	−	−
UICC stage					
I	249 (54.5%)	147 (68.4%)	135 (64.9%)	31 (37.8%)	119 (75.3%)
II	112 (24.5%)	22 (10.2%)	23 (11.1%)	20 (24.4%)	22 (13.9%)
III	63 (13.8%)	30 (14.0%)	24 (11.5%)	26 (31.7%)	13 (8.2%)
IV	25 (5.5%)	16 (7.4%)	8 (3.8%)	5 (6.1%)	2 (1.3%)
Unavailable	8 (1.8%)	−	18 (8.7%)	−	2 (1.3%)
OS					
Alive	289 (63.2%)	52 (24.2%)	98 (47.1%)	43 (52.4%)	124 78.5%)
Dead	156 (34.1%)	163 (75.8%)	110 (52.9%)	39 (47.6%)	34 21.5%)
Unavailable	12 (2.6%)	−	−	−	−
CSS					
Non‐cancer deaths/alive	315 (68.9%)	110 (51.2%)	36 (17.3%)	−	−
Cancer death	89 (19.5%)	105 (48.8%)	74 (35.6%)	−	−
Unavailable	53 (11.6%)	−	98 (47.1%)	−	−
PFS					
No progression	252 (55.1%)	−	132 (63.5%)	48 (58.5%)	−
Progression	116 (25.4%)	−	52 (25.0%)	22 (26.8%)	−
Unavailable	89 (19.5%)	−	24 (11.5%)	12 (14.6%)	−
Follow‐up duration					
Range in months	1–242	1–278	1–159	2–108	2–254
Mean	31.5	92.7	94.7	40.1	86.4
Smoke pack years					
Range in months	0.15–154	1–230	32–224	7–80	−
Mean	41.9	47.3	64.0	46.5	−

Next, using Kaplan–Meier estimates, we show that PATQUANT allows good prognostic stratification of patients when implemented as the IASLC‐proposed grading system (accounting for less than 20% high‐risk patterns in lower grade ranks), shown in Figure 6D , but not reaching statistical significance for differentiating G1 and G2 in multivariate analysis (Table S2). At that, a purely dominant pattern‐based grading, supported by the WHO, (Figure 6E) results in better patient stratification in our analysis (with statistical significance in multivariate analysis when tested together with pT, pN, and patient age; Table S3), raising the question of whether the percentage threshold of high‐risk patterns allowed in G1/G2 can be optimized. Interestingly, Bossé et al. [23] proposed a simplification of the IASLC system through the removal of the complex glandular pattern from a sum of high‐grade patterns, when deciding about G1 and G2 grades. This modification, similar to the WHO system but contrary to the original IASLC system, showed independent prognostic value in multivariate analysis (Figure S20, Table S4). This confirms our observation about the distinct prognostic profile of the complex glandular pattern.

As our tool allows for fully quantitative assessment of pattern abundance, we conducted a systematic evaluation of different cut‐offs (Figures S21–S23) for OS, CSS, and PFS endpoints, showing that higher percentages of high‐risk patterns can still be tolerated in G1/G2, with purely dominant pattern‐based grading (WHO) consistently showing excellent results. For PFS, we showed that the modified IASLC system, with a higher % of high‐grade patterns provided the best results, compared to both the original IASLC system and purely pattern‐based grading (WHO; Figure 6F).

### 2.6 Novel Approaches to Grading Allow for Fine‐Granular Patient Stratification

**FIGURE 7:**
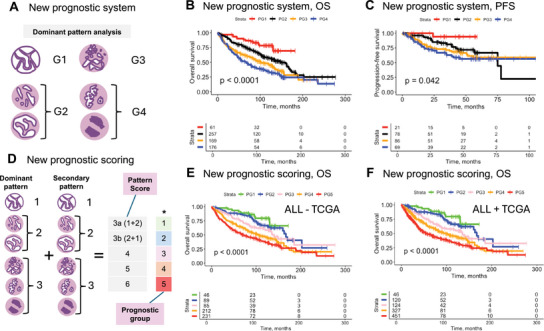
Prognostic study: new approaches to grading. (A) New four‐tier grading system is proposed that accounts for moderate risk profile of the complex glandular pattern resulting in four prognostic grades (G1–G4). (B) New four‐tier grading system. Cohort: merged cohort (PLCO, NLST, UKK, KAMEDA). Endpoint: OS. Univariate/multivariate Cox analysis results in Table S5 showing independent prognostic value when tested together with common clinicopathological variables (pT, pN, age). (C) New four‐tier grading system. Cohort: merged cohort (PLCO, NLST, UKK, KAMEDA). Endpoint: PFS. (D) New prognostic scoring system is proposed, inspired by Gleason grading in prostate cancer. Score is a sum of single grades for dominant and secondary pattern. Score 3 is subdivided into 3a (1 + 2) and 3b (2 + 1). Scoring results in five prognostic groups. The sixth group (*, score 2)—purely lepidic tumors—is not shown due to low relevance in diagnostic routine but might apply in such cases. A more complex system with four‐tier grades for dominant and secondary patterns accounting for moderate risk profile of complex glandular pattern is analyzed in Figure S26, not providing additional benefit. (E) New prognostic scoring system. Cohort: merged cohort (PLCO, NLST, UKK, KAMEDA). Endpoint: OS. Univariate/multivariate Cox analysis results in Table S6 showing a clear independent prognostic value for PG3‐5 and a trend for PG2, not reaching statistical significance likely to low number of observations in PG1 (lepidic predominant tumors); tested together with common clinicopathological variables (pT, pN, age). (F) New prognostic scoring system. Cohort: full merged cohort (TCGA, PLCO, NLST, UKK, KAMEDA); lepidic pattern‐predominant tumors were excluded from TCGA cohort as biased. Endpoint: OS. TCGA cohort was added excluding lepidic‐predominant tumors for which a selection bias was identified (see Results). Univariate/multivariate Cox analysis results in Table S7 showing a clear independent prognostic value for PG3‐5 and a trend for PG2, not reaching statistical significance likely to low number of observations in PG1 (lepidic predominant tumors); tested together with common clinicopathological variables (pT, pN, age). *Comment*: for analysis concerning CSS and PFS endpoints see Figure S24; analysis for pT1‐subcohort in Figure S25. Created in BioRender. Tolkach, I. (2025) https://BioRender.com/q10s060.

Finally, we propose and explore two new approaches to LUAD grading and demonstrate that these enable more fine‐grained stratification of patients with resectable LUAD based on the risk of adverse outcomes (Figure 7).

First, given our previous observation of the more favorable risk profile of the complex‐glandular pattern, compared to classical high‐risk patterns (micropapillary, solid), we test a four‐tier grading system (Figure 7A). We show that in our four‐cohort merged patient collective (excluding TCGA given biases mentioned earlier), this system provides robust risk stratification (Figure 7B,C; Figure S24 for CSS endpoint), which is superior to that of conventional grading approaches (IASLC/WHO) and shows independent added value in multivariate Cox analysis (OS) when combined with common clinicopathological variables (pT, pN, age; Table S7).

Second, inspired by Gleason grading in prostate cancer, we investigate whether the combination of primary and secondary patterns might offer even better patient stratification. We develop a scoring system that results in five prognostic groups (Figure 7D). Notably, this new scoring system enables more fine‐grained patient risk stratification (merged cohort without TCGA; Figure 7E for OS endpoint, Figure S25 for CSS/PFS endpoints), compared to conventional grading approaches (WHO/IASLC). To increase the power of this observation, we conducted an additional validation by merging all five cohorts (patient *n* = 1068) including TCGA but excluding TCGA's lepidic‐predominant cases (which have been shown to be biased, see above; Figure 7F). This analysis showed the same trends as in the reduced cohort. In univariate and multivariate Cox analysis for both cohorts (ALL‐TCGA: Table S6; ALL+TCGA: Table S7), a clear independent prognostic value was evident for PG3‐5, while PG2 showed a statistical trend likely not reaching statistical significance due to the low number of observations/events in PG1. For the analysis of more complex version of the new scoring system, which includes the complex‐glandular pattern as a separate scoring tier, see Figure S27. This modification appears to be too complex and does not bring an additional benefit still resulting in five prognostic groups. Although both new four‐tier grading and five‐tier scoring system show excellent prognostic performance, the five‐tier system would be preferable, given more fine‐grained risk stratification.

### 2.7 Analysis of Prognostic Value in Stage I and pT1 Tumors

In addition, we evaluated the prognostic value of the WHO/IASLC grading systems, the new four‐tier grading system, and the new five‐tier scoring system (as implemented in PATQUANT) in patients with Stage I and pT1 tumors. Overall survival (OS) was selected as the primary endpoint for this subcohort, as fewer events were observed in the cancer‐specific survival (CSS) and progression‐free survival (PFS) endpoints. Both the WHO and IASLC grading systems demonstrate prognostic values in pT1 (Figure S20A,B) and Stage I tumors (Figure S20C,D; uni‐ and multivariate analysis in Tables S8 and S9), similar to results from the all‐stage cohort.

Notably, the new four‐tier prognostic system maintains strong prognostic capabilities when applied to the pT1 (Figure S24A) and Stage I (Figure S24B; uni‐ and multivariate analysis in Table S10) subgroups. Additionally, the new five‐tier prognostic scoring shows prognostic performance comparable to that of the all‐stage cohort in both Stage I (Figure S24A ; uni‐ and multivariate analysis in Table S11) and pT1 subcohorts (Figure S24B–D).

## 3 Discussion

In our study, we developed PATQUANT, a powerful AI tool for fully automated and quantitative histological assessment of LUAD patterns in H&E‐stained WSIs. We demonstrated that the developed tool outperformed 8 out of 13 experienced pathologists in large interobserver agreement study and performed exceptionally well as a prognostic tool independent of grading implementation (WHO/dominant pattern‐based, IASLC with high‐grade pattern thresholding). Pathologists assisted by PATQUANT showed twice higher interobserver agreement. Moreover, we showed that fully quantitative nature of PATQUANT allows for the extraction of more granular prognostic information from patterns, which we implemented as two new grading approaches, including Gleason grading‐inspired prognostic scoring from both the dominant and secondary pattern.

LUAD patterns are a main component of the modern grading systems, with a clearly recognized prognostic value [2, 3]. Although the WHO classification still recommends using five common patterns (lepidic, acinar, papillary, micropapillary, and solid) and a three‐tier grading based on the dominant pattern [2], there is clear evidence that one additional pattern can be separated from the acinar type (complex glandular pattern: cribriform and fused glands) having a higher risk profile and negative prognostic implications [3, 4, 5, 6]. Moreover, the IASLC‐proposed grading system recommends classifying G1 and G2 tumors as G3 if high‐grade patterns (complex glandular, micropapillary, and solid) constitute more than 20% of the tumor, which has been shown to improve risk stratification capabilities to a certain extent [3]. For that, the authors do not implement multivariate Cox proportional hazard analysis with typical clinicopathological variables and use a test cohort of 300 cases.

In our study, for the annotations of the training dataset, we used Moreira et al. [3] description of complex glandular pattern as a reference. Importantly, in our highly representative, large, multi‐institutional, and multi‐national cohort of patients with resectable LUAD, we made an important observation concerning complex glandular pattern. We demonstrated that this pattern carries a moderate prognostic risk, positioned between low‐risk patterns (acinar, lepidic) and high‐risk ones (micropapillary, solid), with the lepidic pattern being of very low risk (Figure 6A–C). Notably, the four‐tier grading system, which accounts for this (dominant pattern‐based grading; Figure 7A,C,D), appears to provide more effective risk stratification than the classical three‐tier grading systems (WHO or IASLC; Figure 6D–F).

In our large interobserver agreement study, we further show that pathologists and the AI tool detection exhibit high levels of agreement in detecting this pattern, allowing for robust recognition (Figure 3B,C). Thus, pathologists reached consensus on the complex glandular pattern being dominant in 55/62 ROIs (Figure 3B), and the AI was highly concordant with the majority vote results for this pattern (Figure 3C).

Moreover, we conducted a detailed analysis using different thresholds for high‐grade patterns in the IASLC grading approach (Figures S21–S23) and concluded that predominant‐pattern‐based grading (the WHO‐recommended approach) provides better prognostic stratification of patients, at least with the developed tool that was validated to have very high pixel‐wise accuracy for pattern detection (Figure 2), outperforming the majority of pathologists in a comprehensive agreement study (Figure 3). Given these validation results, a question arises as to whether the abundance of high‐grade patterns in lower‐grade cases should be strictly accounted for during grading. There is still an ongoing discussion about an optimal grading system, with several studies showing in general a reasonable, sometimes comparable prognostic performance for both IASLC‐proposed and dominant‐pattern based systems [23, 24, 25, 26, 27]. Some authors proposed simplification of the IASLC system through removal of complex glandular pattern from high‐grade pattern definition [23], which could facilitate reproducibility. Our results provide evidence that complex glandular pattern has a distinct prognostic profile and should be accounted for during grading (Figures 6A and 7A,C,D).

We propose and implement a new scoring system using PATQUANT, inspired by Gleason grading, which accounts for dominant and secondary patterns (Figure 7). This system should be regarded as a further refinement of the scoring concept introduced by Sica et al. [28]. Different to Sica et al. and similar to Gleason grading, we subdivide Score 3 into 3a (1 + 2) and 3b (2 + 1), resulting in five prognostic groups (Figure 7B) that provide excellent, fine‐grained prognostic stratification, superior to any classical grading systems. This system is easy to implement, even without AI support, and should be considered as a promising candidate for LUAD grading, pending independent validation. Interestingly, a similar but more fine‐grained scoring system (which considers the complex glandular pattern as a separate, intermediate‐risk pattern) seems to be overly complex and does not provide additional benefit (Figure S27).

All tested grading implementations (IASLC, WHO, new four‐tier grading system, new five‐tier scoring system) show the prognostic performance in Stage I and pT1 tumor similar to the results from the all‐stage cohort.

A newly developed PATQUANT represents a powerful tool for pattern quantification and downstream grading applications. Several other AI‐based tools for pattern analysis have been reported. Lami et al. [17] developed a classification tool for patch‐level detection of patterns. This tool has several limitations, such as its large patch size (1 mm^2^ patches vs. pixel‐wise precision in our tool), which allows only coarse tumor mapping, and underrepresentation of complex glandular pattern (0.4% of all patches). The study performed a limited prognostic validation with a cohort of 141 patients from a single center. Pan et al. [21] trained a segmentation tool (ANORAK) that accounts for complex glandular pattern and extensively, successfully validated it using multi‐institutional cohort of 1372 patient cases, including agreement analysis with three pathologists and assessment prognostic value. For the latter, the authors implemented the IASLC grading system.

PATQUANT has several advantages over ANORAK. We used a manual annotation area at least 100 times larger, derived from 177 annotated slides across multiple departments, compared to 49 WSIs in Pan et al. [21]. This difference is likely reflected in the significantly lower segmentation accuracy for ANORAK, recognized by authors themselves (Dice scores range for patterns 0.44–0.74, compared to 0.77–0.94 for our tool). Our pipeline includes a highly precise, independently validated tumor detection/segmentation backbone [14], which is of utmost importance for downstream pattern analysis. Moreover, we demonstrate that our new scoring approaches provide significantly more granular risk stratification of patients, superior to both the WHO and IASLC grading system. An additional strength of our study is a large‐scale interobserver agreement study involving 13 pathologists from 5 countries. Several other studies have developed DL models for pattern detection, including one for mouse models, but these models generally have significant limitations, such as lacking adequate prognostic validation and failing to account for all six patterns [15, 16, 18, 19, 20].

Our study is not without limitations. We used retrospective patient cohorts for validation, and therefore, prospective validation is warranted. The superior prognostic performance of our new scoring systems should be confirmed using independent patient cohorts. Our prognostic cohorts are underpowered for lepidic‐predominant tumors, which might be responsible for lacking statistical significance in some of multivariate tests; moreover, we found that the TCGA cohort is biased through the selection of slides with certain target characteristics.

## 4 Conclusion

In this study, we developed a powerful, fully automated AI tool for LUAD pattern detection and quantification (PATQUANT). PATQUANT outperformed 8 out of 13 experienced pathologists in a comprehensive interobserver agreement analysis. Using a large multi‐national patient cohort, we validated the prognostic capabilities of PATQUANT and proposed two new, reproducible scoring systems that allow for a fine‐ grained stratification of prognosis in patients with resectable LUAD. These might be a foundation for more personalized selection of therapies in patients with LUAD and might be used in course of clinical studies for development of new therapy approaches. We are also releasing publicly our large agreement dataset with pathologists’ scores for educational purposes and to facilitate further algorithm development and validation in this domain.

## 5 Materials and Methods

### 5.1 Patient Cohorts and Digitization

Five patient cohorts with resectable LUAD were included in this study (Figure 1D, detailed clinicopathological characteristics are presented in Table 1): The Cancer Genome Atlas (TCGA; international) LUAD cohort consisting of patients from multiple institutions (*n* = 36), the National Lung Screening Trial (NLST; United States of America/USA) cohort, the Prostate, Lung, Colorectal, and Ovarian Cancer Screening Trial (PLCO; USA) cohort, the University Hospital of Cologne (UKK; Germany; 2014–2020) cohort, and the Kameda Medical Center (KAMEDA; Japan; 2014–2020) cohort. The cohorts represent a broad range of clinical and pathological stages. Inclusion criteria were neoadjuvant‐therapy‐naïve status, availability of digitized histological sections (hematoxylin & eosin‐stained), and follow‐up information (for prognostic study). Exclusion criteria were invasive mucinous carcinoma (pattern grading not applicable) and adenosquamous carcinoma. Information about overall survival (OS), cancer‐specific survival (CSS), or progression‐free survival (PFS) endpoints was available, depending on the cohort (Figure 1D). Information on the digitization of histological slides is presented in Figure 1D. The WSIs were digitized with at least 20x magnification, corresponding to a micron‐per‐pixel (MPP) parameter of approximately 0.5 or lower. The slides were controlled for quality using both automated tools and manual review.

### 5.2 Training Dataset and Manual Annotations

The WSIs of 177 patients from TCGA cohort were used for training purposes, selected manually to represent a broad range of morphological patterns and significant inter‐institutional/lab heterogeneity. On all slides, several (1–5) representative regions were selected for manual annotations, which were performed by two board‐certified pathologists (YT, YW), both having substantial experience in lung cancer pathology. Regions with discrepant categorizations were discussed, and a consensus was reached on the pattern. A substantial part of the regions selected for manual annotations contained more than one pattern. All pattern annotations were made with pixel‐wise precision and of very high quality in QuPath v. 0.4.3 and higher [29] (Figure 1C); annotations of tumor tissue as a class were inherited from previous work [14]. Six patterns were annotated, according to recommendation of IASLC (Figure 1C) [3].

### 5.3 Algorithm Development

Annotated regions were extracted in the form of patches of size 512 × 512 px at MPP 1.0 (roughly 10x objective magnification) with an overlap of 200 px. Annotations were exported as masks with class coding, including class “0” for non‐annotated regions. Patches with less than 5% of annotated area were ignored. Background pixels were removed from annotated pattern regions using a previously trained model [30]. The resulting dataset contained 38,392 unique patches, of which approximately 10% were reserved for validation/fine‐tuning during training (case‐level split). Algorithm construction was performed using segmentation‐models‐pytorch v.0.3.1 package and using training pipeline developed in previous studies [14, 30]. We treat all pipeline components—encoder architecture, decoder architecture, batch size, and weighting strategy—as hyperparameters and systematically evaluate them on the validation dataset. For most histological tasks, we have repeatedly shown that 10× magnification yields the best results; therefore, we employ it in this study [14, 30]. Furthermore, a 512‐pixel patch size has consistently proven to be the optimal choice for semantic segmentation in pathology [14, 30]. UNet, Unet++, DeepLabv3+ decoder architectures were used for training, with EfficientNet and ResNet family encoders, which were completely re‐trained using training dataset. UNet++ with EfficientNetB0 encoder showed better results during validation and was selected for final implementation. Categorical cross‐entropy loss and the Adam optimizer with initial learning rate (LR) of 0.0005 were applied, with 10x decay after eighth epoch and occurring every 4 epochs. The Albumentations package was used for data augmentation (rotations, flips, Gaussian noise, jpeg compression, random brightness, gamma, contrast, hue, saturation) with probability of each augmentation time set at approximately 30%. Batch balancing was implemented with and without weight correction, with oversampling of the overrepresented classes to match the number of patches in the dominant class shown best results. Training was performed in batches of 6, 12, and 18 with a batch size of 18 showing the best results during validation. The PyTorch 1.10 (Python 3.9) framework was used for development and inference.

### 5.4 Model Inference and WSI Pipeline

The model is applied to regions detected as tumor and is integrated with a non‐small cell lung cancer (LUAD, lung squamous cell carcinoma) detection tool that was developed and extensively validated previously [14]. The entire pipeline consists of three algorithms: tissue detection (1x magnification analysis), tumor detection (10x magnification analysis), and pattern subtyping algorithms (10x magnification analysis). The average analysis time for the complete pipeline is 2–6 min (depending on the number of tissue sections) for resection cases. Pattern abundance is quantified at the pixel level, that is, from regions that were initially detected as tumor and later classified as patterns. Therefore, we account for the fact that typically in the same region, epithelial tumor component will be more abundant in higher grade patterns, compared to lower grade patterns, especially lepidic, and use correction weights derived from distribution in training dataset for pattern area in ROI or WSI (lepidic: 1.41, acinar: 1.35, papillary: 1.06, micropapillary: 1.14, solid: 1.0, complex glandular: 1.10).

### 5.5 Test Dataset: Formal Validation

One independent dataset (UKK) with 20 LUAD resection cases (WSI *n* = 20; H&E‐stained), representing a broad spectrum of patterns, was manually annotated with high precision (using principles similar to those outlined above for the training dataset). This dataset was used for pixel‐level formal statistical validation of the segmentation accuracy of the pattern algorithm and for pixel‐level analysis of misclassifications between different patterns.

### 5.6 Test Dataset: Interobserver Agreement Analysis

A dataset consisting of large regions of interest (ROIs) from multiple cases across three international cohorts of resection cases (Germany: UKK, Japan: KAMEDA, USA: cohort of the National Cancer Institute's Clinical Proteomic Tumor Analysis Consortium/CPTAC) was created (*n* = 283). This dataset is representative of three countries (accounting for variations in LUAD tumor genetics), different laboratory practices, and scanners (UKK: Leica GT450, KAMEDA: Philips, CPTAC: Leica AT2 and older models). The examples are presented in Figure 4.

### 5.7 Pathologists

Thirteen pathologists specializing in lung cancer from five countries were included in the study. All of them provided their pattern analysis for 283 ROIs of interobserver agreement analysis dataset.

### 5.8 Data Analysis and Statistics

Data analysis and plotting were performed in R v. 4.4.0. Agreement heatmaps were created using the seaborn package for Python. Kappa statistics with equal weights as well as simple agreement were used for analysis of interobserver agreement. Additionally majority voting and leave‐one‐out approach were used to compare the dominant pattern provided by single pathologists. Majority vote is the most frequent dominant pattern provided by single pathologists (excludes AI). If 7 and more pathologists provided the same grading, then consensus dominant pattern could be reached. In the leave‐one‐out analysis—to exclude the influence of single pathologists on the majority vote—we excluded these single pathologists from majority vote calculation (e.g., Pathologist 1 vs. majority vote results Pathologists 2–13). Kaplan–Meier estimates with long‐rank test as well as univariate and multivariate Cox proportional hazard models including common clinicopathological variables were implemented. OS, CSS, and PFS were used as endpoints. TRIPOD‐AI Checklist is provided as Supplementary Data.

### 5.9 Hardware

The algorithm training was performed on the high‐performance cluster of the University of Cologne equipped with 12x NVIDIA V100 32Gb graphic cards. All tests and large‐scale inference in prognostic cohorts were performed on the AI server with 4x A100 80Gb NVIDIA cards.

## Author Contributions

Y.W. was responsible the data preparation, annotation, data management, data analysis, and manuscript drafting. K.L. prepared and analyzed the data. M.H. managed the clinical data. W.A. prepared the data. S.S., A.B., Y.Y., D.J., X.Z., S.C., A.S., A.Q., A.R., J.F., and A.L.M. analyzed the data. R.B. analyzed the data, supervision, and resources. Y.T. drafted the study concept and design, data preparation, data annotation, algorithm training and validation, data analysis, manuscript drafting, resources, and supervision. All authors performed the critical revision for important intellectual content. All authors have read and approved the final manuscript.

## Ethics Statement

All study steps were performed in accordance with the Declaration of Helsinki. This study was approved by the Ethical committees of the University of Cologne (22‐1233, 20‐1583) and Kameda General Hospital (22‐094). The need for patient consent was waived as only anonymized, retrospective archive materials were used (no involvement of human participants).

## Conflicts of Interest

Author Sofia Campelos is an employee of IMP Diagnostics but has no relevant financial or non‐financial interests to disclose. The other authors declare no conflicts of interest.

## Supporting information




**Supplementary File 1**: mco270380‐sup‐0001‐SupMat.pdf

## Data Availability

TCGA, PLCO, and NLST datasets are publicly available. An interobserver agreement dataset with results of evaluation by pathologists and Python code used for algorithm training and WSI analysis is available at Zenodo for academic, non‐commercial use only (https://zenodo.org/records/15512151). The code is also available as part of GitHub repositorium (https://github.com/cpath‐ukk/lung_cancer). Full versions of datasets are available from the corresponding author upon reasonable request.
