# Supplementary material for: AI Algorithm for Lung Adenocarcinoma Pattern Quantification (PATQUANT): International Validation and Advanced Risk Stratification Superior to Conventional Grading
Source: MedComm (2020). 2025 Sep 8;6(9):e70380. doi: 10.1002/mco2.70380 (PMC12415442; doi:10.1002/mco2.70380)
Supplement: Supplementary file 1 — mco270380‐sup‐0001‐SupMat.pdf [file MCO2-6-e70380-s001.pdf]

AI algorithm for lung adenocarcinoma pattern quantification (PATQUANT): International validation and advanced risk stratification superior to conventional grading

Yuan Wang[1], Kris Lami[2], Waleed Ahmad[1], Simon Schallenberg[3], Andrey Bychkov[2,4], Yuanzi Ye[5], Danny Jonigk[6,7], Xiaoya Zhu[8], Sofia Campelos[9], Anne Schultheis[10], Matthias Heldwein[11], Alexander Quaas[1], Ales Ryska[12], Andre L. Moreira[13], Junya Fukuoka[2,4], Reinhard Büttner[1*], Yuri Tolkach[1*]

[1]Institute of Pathology, University Hospital Cologne, Cologne, Germany
[2]Department of Pathology Informatics, Nagasaki University, Nagasaki, Japan
[3]Institut für Pathologie, Charité, Berlin, Germany
[4]Department of Pathology, Kameda Medical Center, Kamogawa, Japan
[5]Department of Pathology, The First Affiliated Hospital of Anhui Medical University, Hefei, China
[6]Institute of Pathology, University Hospital Aachen, Aachen, Germany
[7]German Center for Lung Research, DZL, BREATH, Hanover, Germany
[8]Department of Pathology of Run Run Shaw Hospital affiliated to Zhejiang University, Hangzhou, China
[9]IMP Diagnostics, Porto, Portugal
[10]Institute of Pathology, University Hospital Freiburg, Freiburg, Germany
[11]Department of Cardiothoracic Surgery, University Hospital Cologne, Germany
[12]The Fingerland Department of Pathology, Charles University Medical Faculty Hospital, Hradec Králové, Czech Republic
[13]Department of Pathology, NYU Grossman School of Medicine, New York, NY, USA

*Co-senior authors.

*Correspondence:
Yuri Tolkach MD PhD
Institute of Pathology
University Hospital Cologne
Cologne, Germany
Phone: +49 221 478 6365
Fax: +49 221 478 6360
Email: yuri.tolkach@gmail.com

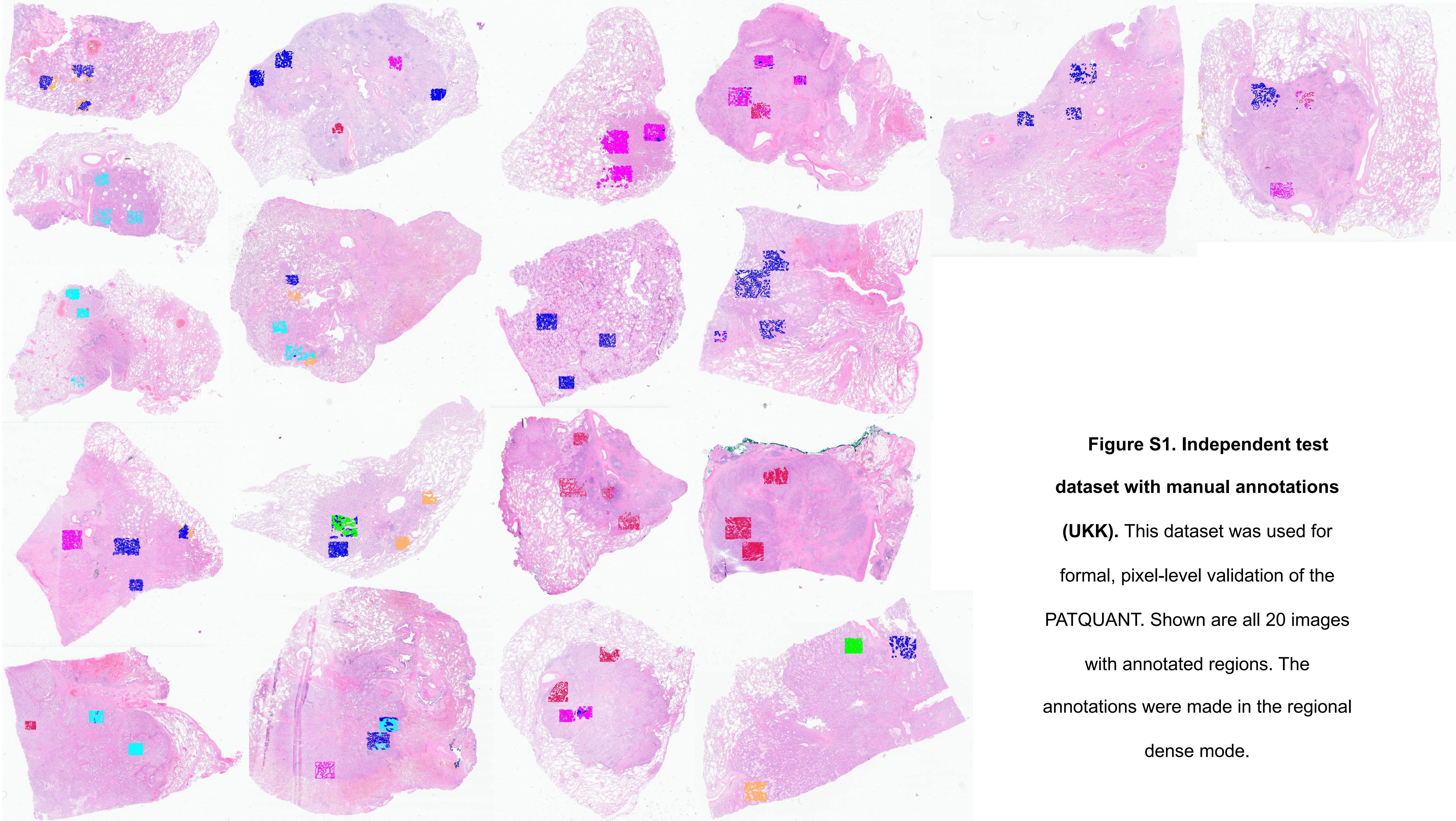

Figure S1. Independent test dataset with manual annotations (UKK). This dataset was used for formal, pixel-level validation of the PATQUANT. Shown are all 20 images with annotated regions. The annotations were made in the regional dense mode.

Interobserver agreement analysis: Simple agreement

	AI	P1	P2	P3	P4	P5	P6	P7	P8	P9	P10	P11	P12	P13
**P13**	47	43.46	52.3	55.12	57.95	62.9	58.3	41.34	49.47	61.84	42.05	61.13	48.41	100
P12	64.31	63.96	55.12	51.59	53.36	59.01	53.71	54.77	66.78	60.42	56.18	52.65	100	48.41
P11	51.59	49.47	59.01	58.66	59.72	73.5	58.66	44.52	55.12	67.49	51.94	100	52.65	61.13
P10	56.89	55.12	50.88	44.88	53.71	58.66	56.89	45.23	69.61	56.89	100	51.94	56.18	42.05
P9	60.07	53.71	56.18	55.48	63.6	71.38	67.14	45.23	62.9	100	56.89	67.49	60.42	61.84
P8	60.07	66.43	59.72	49.47	60.42	60.42	60.78	50.88	100	62.9	69.61	55.12	66.78	49.47
P7	47.35	61.48	62.9	39.93	44.88	47.35	42.4	100	50.88	45.23	45.23	44.52	54.77	41.34
P6	50.53	47.35	46.64	54.77	59.36	60.78	100	42.4	60.78	67.14	56.89	58.66	53.71	58.3
P5	56.18	54.42	60.42	58.3	64.31	100	60.78	47.35	60.42	71.38	58.66	73.5	59.01	62.9
P4	50.88	54.06	54.77	54.06	100	64.31	59.36	44.88	60.42	63.6	53.71	59.72	53.36	57.95
P3	47.35	39.93	44.17	100	54.06	58.3	54.77	39.93	49.47	55.48	44.88	58.66	51.59	55.12
P2	50.88	62.9	100	44.17	54.77	60.42	46.64	62.9	59.72	56.18	50.88	59.01	55.12	52.3
P1	56.89	100	62.9	39.93	54.06	54.42	47.35	61.48	66.43	53.71	55.12	49.47	63.96	43.46
**AI**	100	56.89	50.88	47.35	50.88	56.18	50.53	47.35	60.07	60.07	56.89	51.59	64.31	47

Agreement: 100, 90, 80, 70, 60, 50, 40

Mean simple agreement	P7	P3	P13	P10	AI	P1	P2	P6	P4	P12	P11	P8	P9	P5
	48%	50%	52%	54%	54%	55%	55%	55%	56%	57%	57%	59%	60%	61%

Figure S2. Interobserver agreement study: simple agreement analysis. Confusion matrix showing systematic intergarder agreement comparisons using simple agreement metrics. For kappa statistics refer to Figure 3. Simple agreement does not account for agreement by chance only, while kappa statistics considers for this constellation.

A Influence of PATQUANT AI-assistance on interobserver agreement: kappa metrics

Without assistance (initial experiment)

	P6	P12	P13
P6		**0.37**	**0.40**
P12	**0.37**		**0.36**
P13	**0.40**	**0.36**	

With assistance (extended experiment)

	P6	P12	P13
P6		**0.67**	**0.78**
P12	**0.67**		**0.74**
P13	**0.78**	**0.74**	

B Influence of PATQUANT AI-assistance on interobserver agreement: heatmap

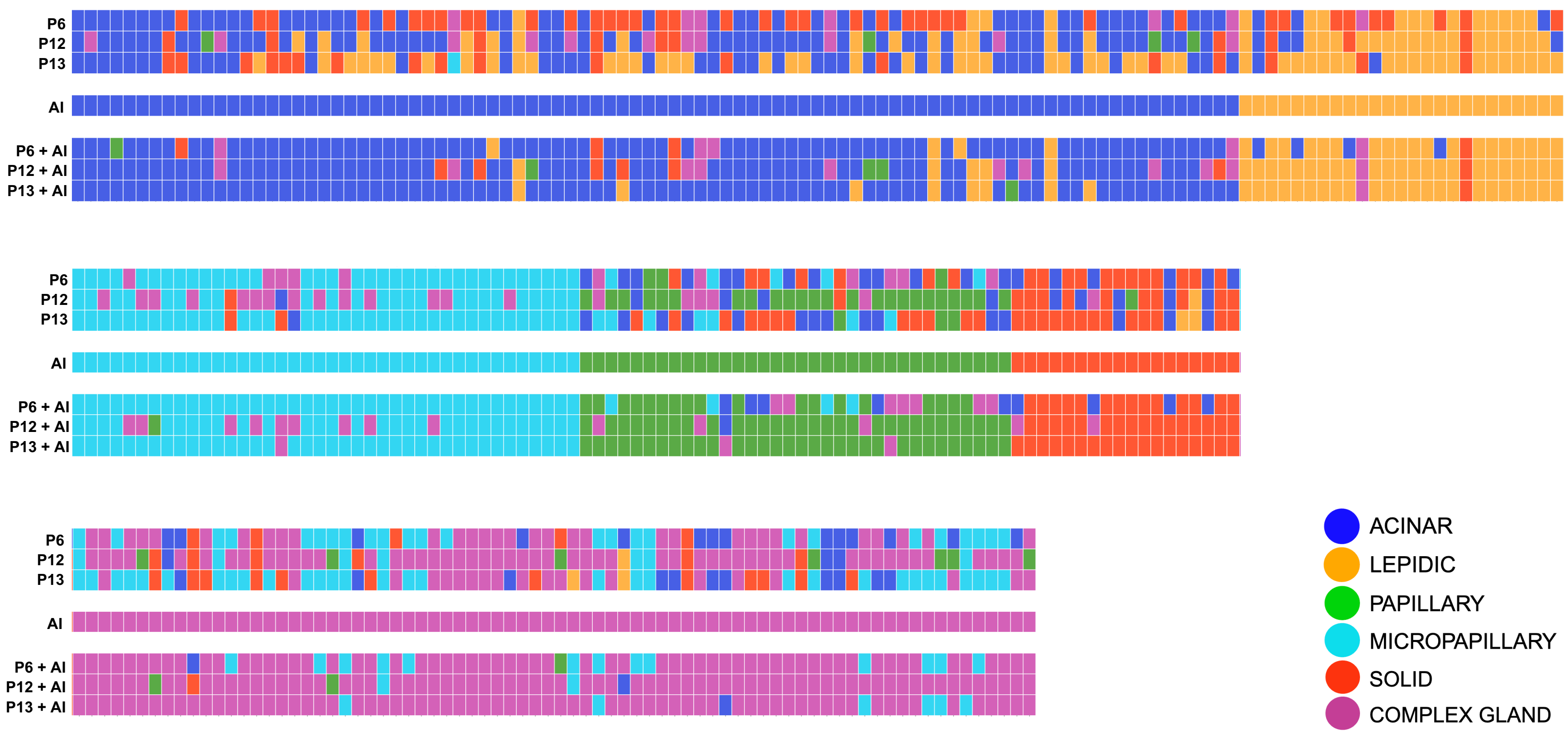

Figure S3. Comparison of interobserver agreement for pattern subtyping (a dataset of regions of interest, n=283) with and without AI-assistance by developed PATQUANT tool. A. Three pathologists (P6, P12, and P13) evaluated dataset firstly without AI-assistance (table above) showing equally weighted kappa levels of maximally 0.40 (dominant pattern assessment). After wash-out period (9 months) the pathologists re-evaluated the same regions once more time with AI assistance showing kappa levels of up to 0.78. B. Heatmap of dominant pattern distribution for single analyzed images: upper part – initial evaluation, middle part – evaluation by AI, lower part – pathologists assisted by PATQUANT.

WSI pipeline: example 1 of WSI processing

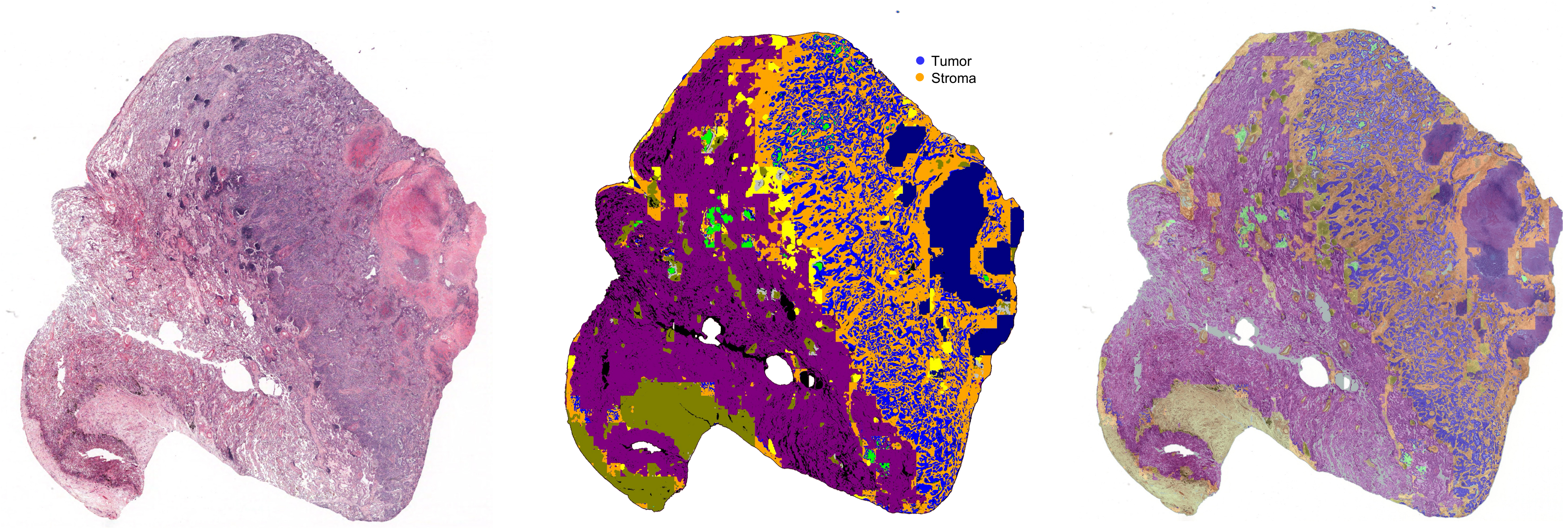

Figure S4. Example of inference using PATQUANT whole-slide image pipeline. Shown in detail is the whole-slide image presented in Figure 5A. Left side: original Hematoxylin&Eosin-stained slide. Middle: segmentation mask of the multi-class tissue segmentation algorithm developed earlier. Blue is tumor, orange is tumor stroma with other colors depicting different other tissue classes. Right side: overlay of multi-class tissue segmentation mask on the original image.

Figure S5. Example of inference using PATQUANT whole-slide image pipeline. Shown in detail is the whole-slide image presented in Figure 5A. Left side: segmentation mask of six patterns from PATQUANT. Right side: original Hematoxylin&Eosin-stained slide.

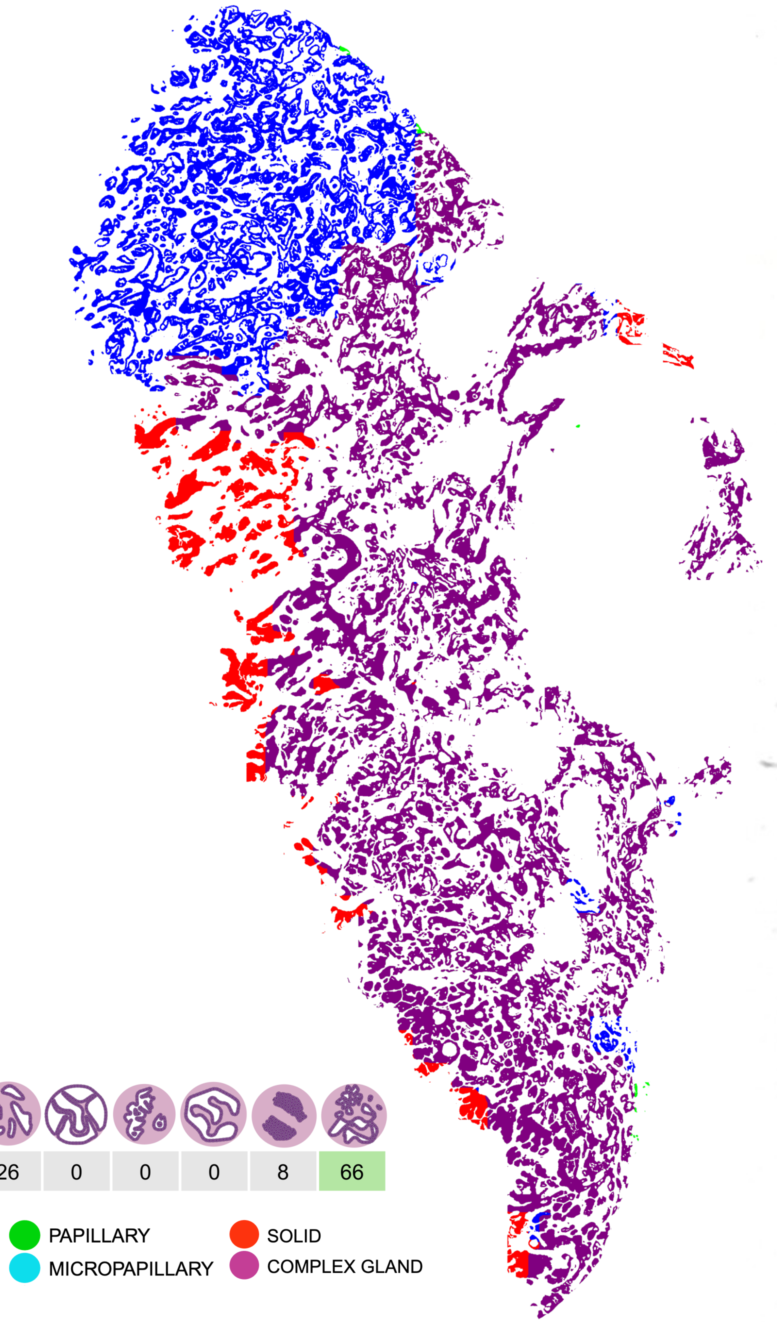

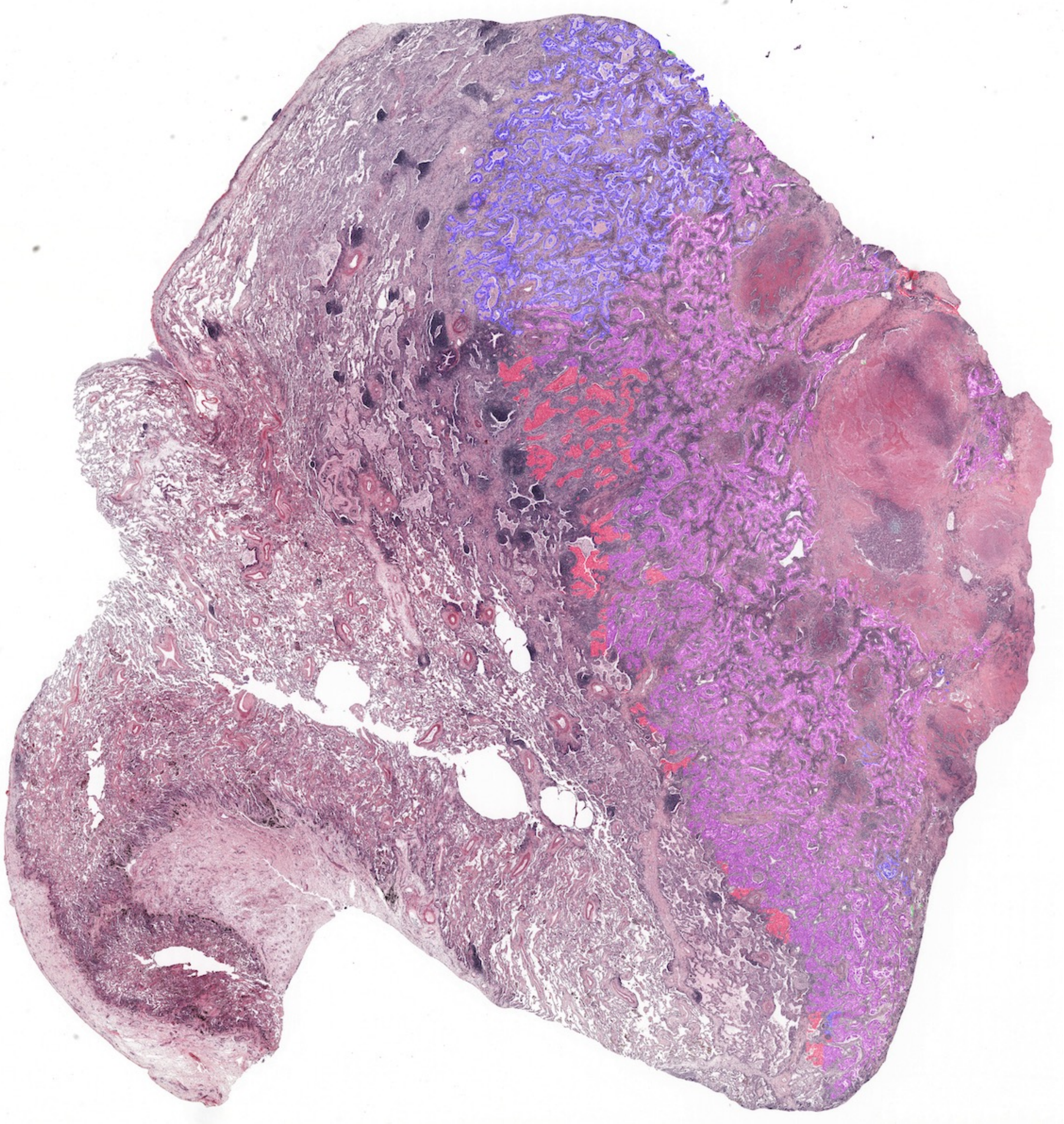

Created in BioRender. Tolkach, I. (2025) https://BioRender.com/q10s060

WSI pipeline: example 2 of WSI processing

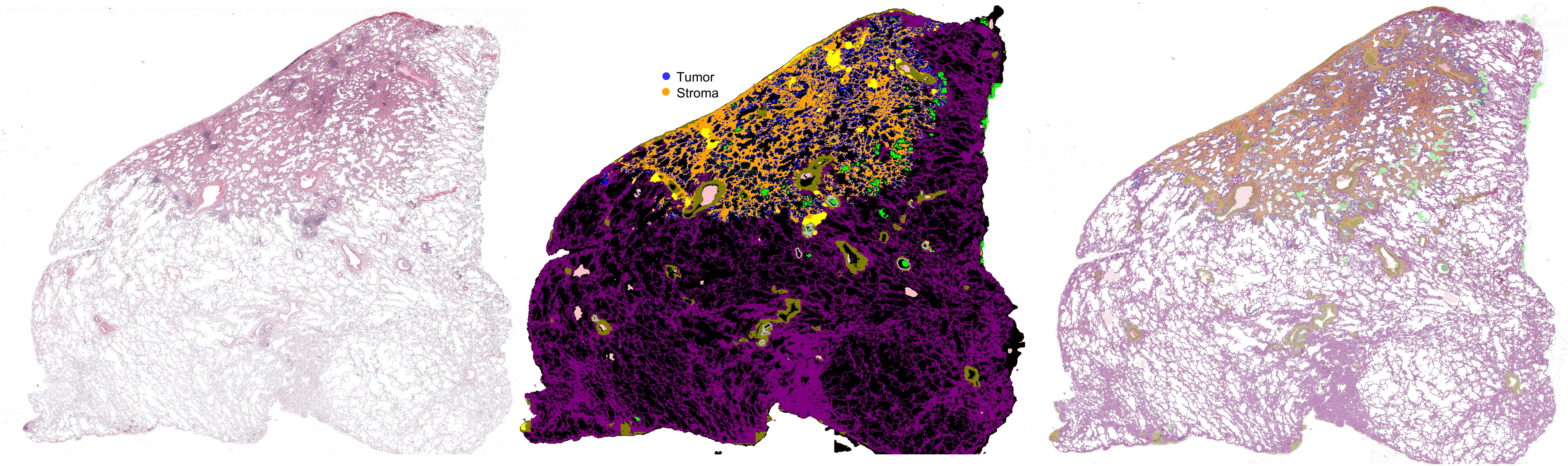

Figure S6. Example of inference using PATQUANT whole-slide image pipeline. Shown in detail is the whole-slide image presented in Figure 5B. Left side: original Hematoxylin&Eosin-stained slide. Middle: segmentation mask of the multi-class tissue segmentation algorithm developed earlier. Blue is tumor, orange is tumor stroma with other colors depicting different other tissue classes. Right side: overlay of multi-class tissue segmentation mask on the original image.

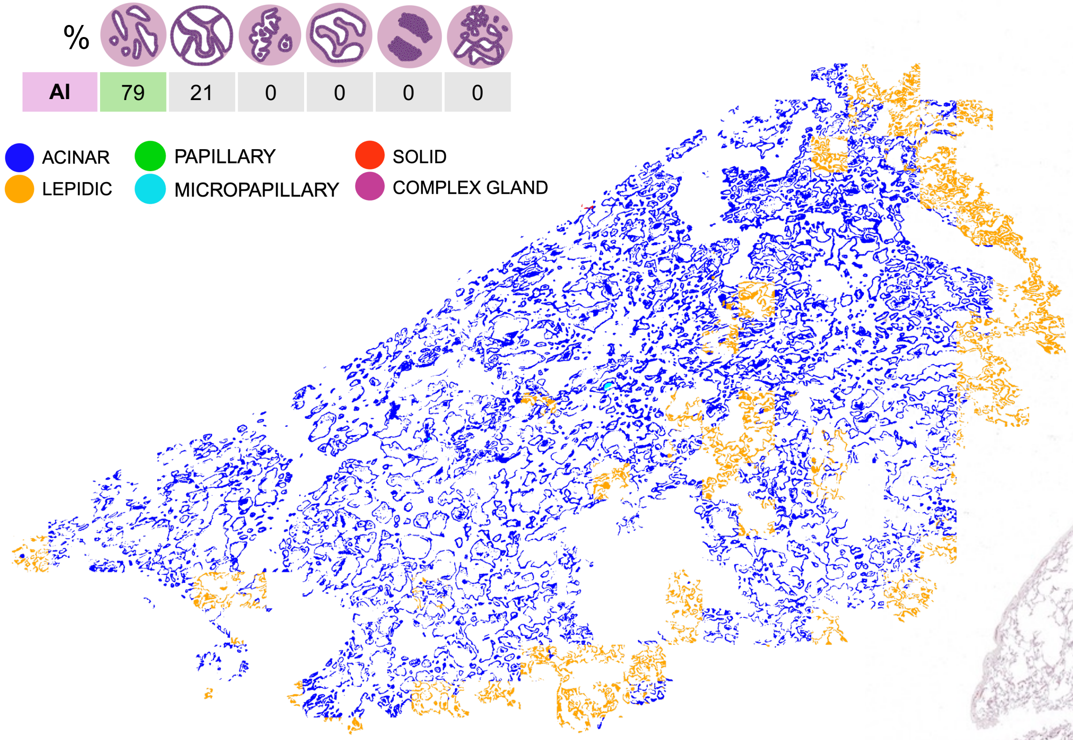

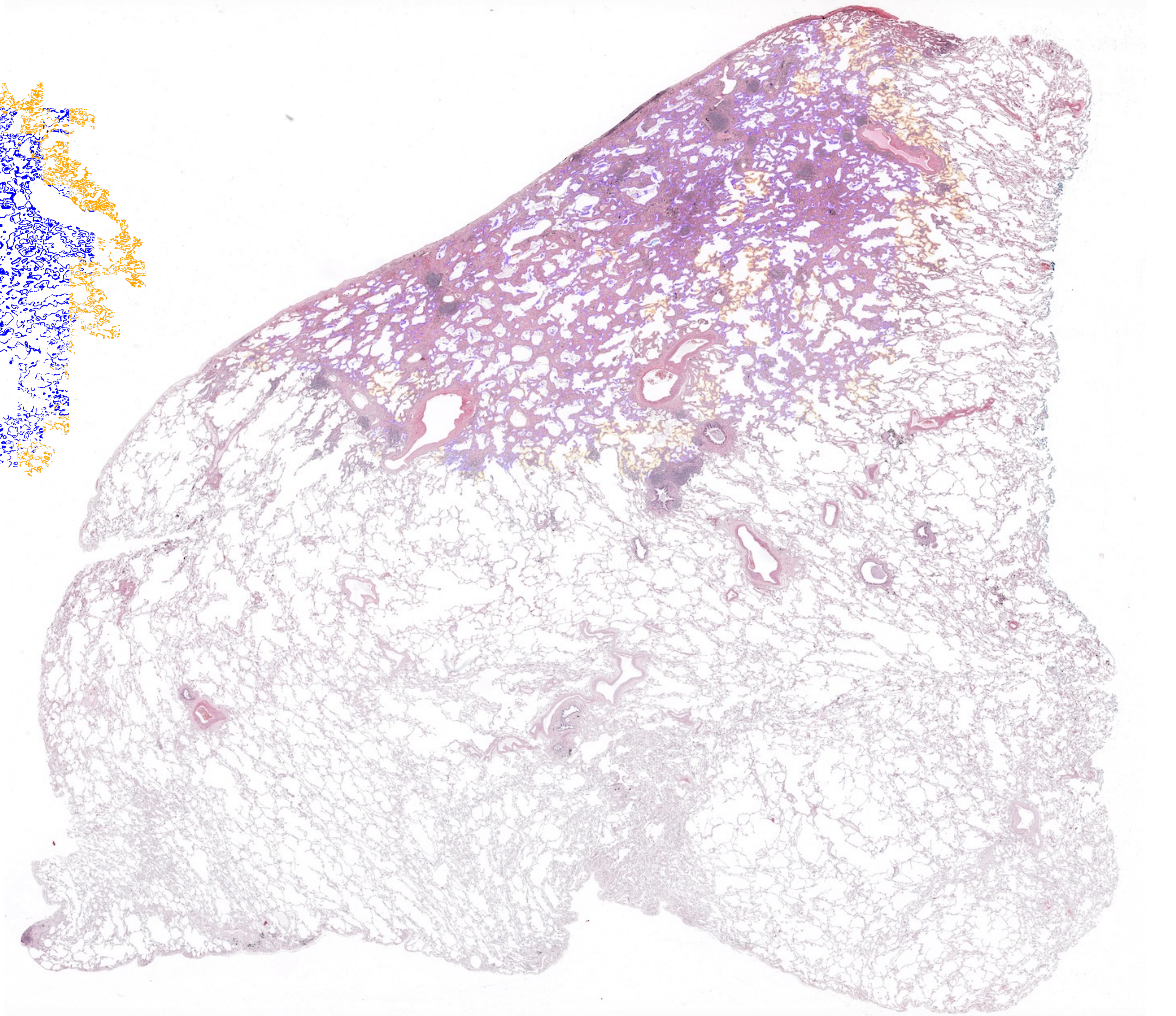

Figure S7. Example of inference using PATQUANT whole-slide image pipeline. Shown in detail is the whole-slide image presented in Figure 5B. Left side: segmentation mask of six patterns from PATQUANT. Right side: original Hematoxylin&Eosin-stained slide.

Created in BioRender. Tolkach, I. (2025) https://BioRender.com/q10s060

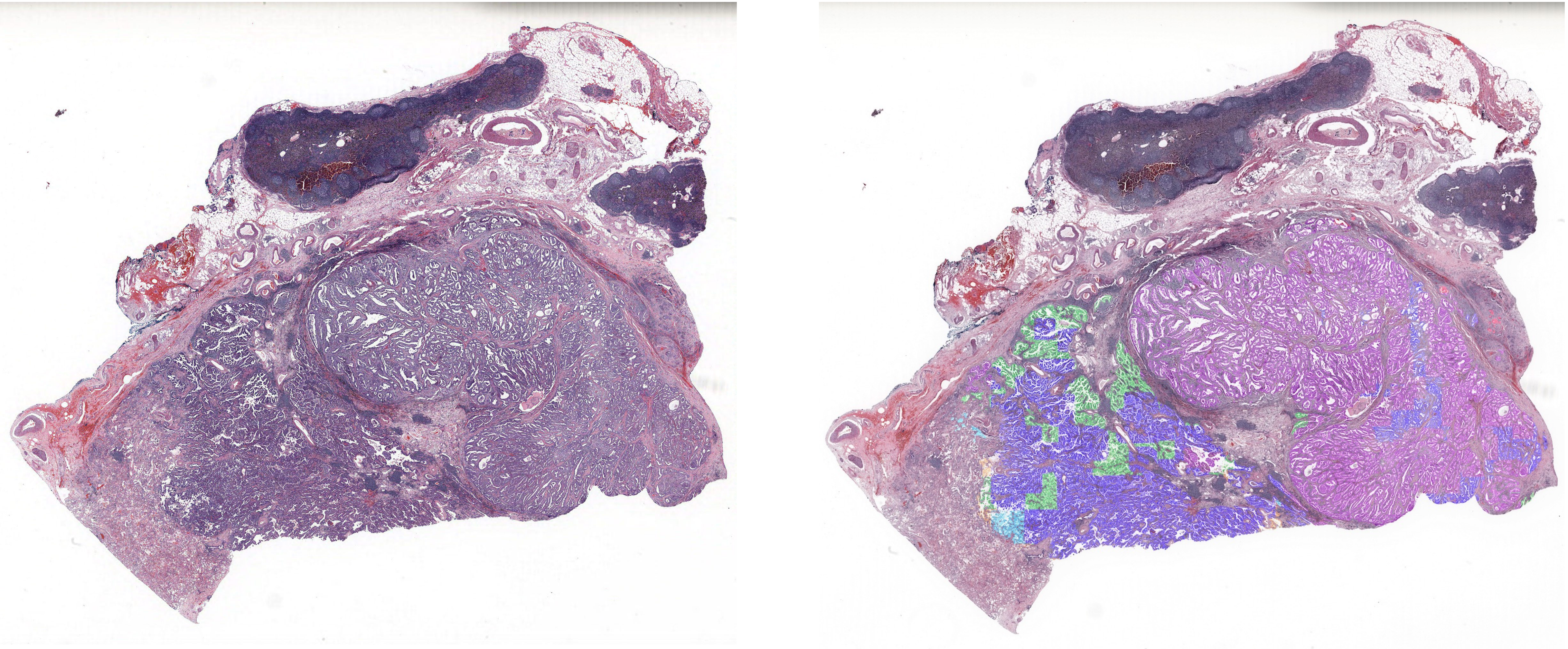

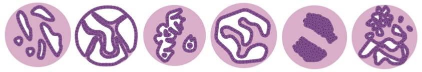

%						
AI	26	0	2	8	0	64

ACINAR
PAPILLARY
SOLID
LEPIDIC
MICROPAPILLARY
COMPLEX GLAND

Figure S8. Further example of whole-slide image inference using PATQUANT.

Metrics are provided. Left side: original H&E image. Right side: overlay of PATQUANT pattern segmentation map on the original H&E image.

Created in BioRender. Tolkach, I. (2025) https://BioRender.com/q10s060

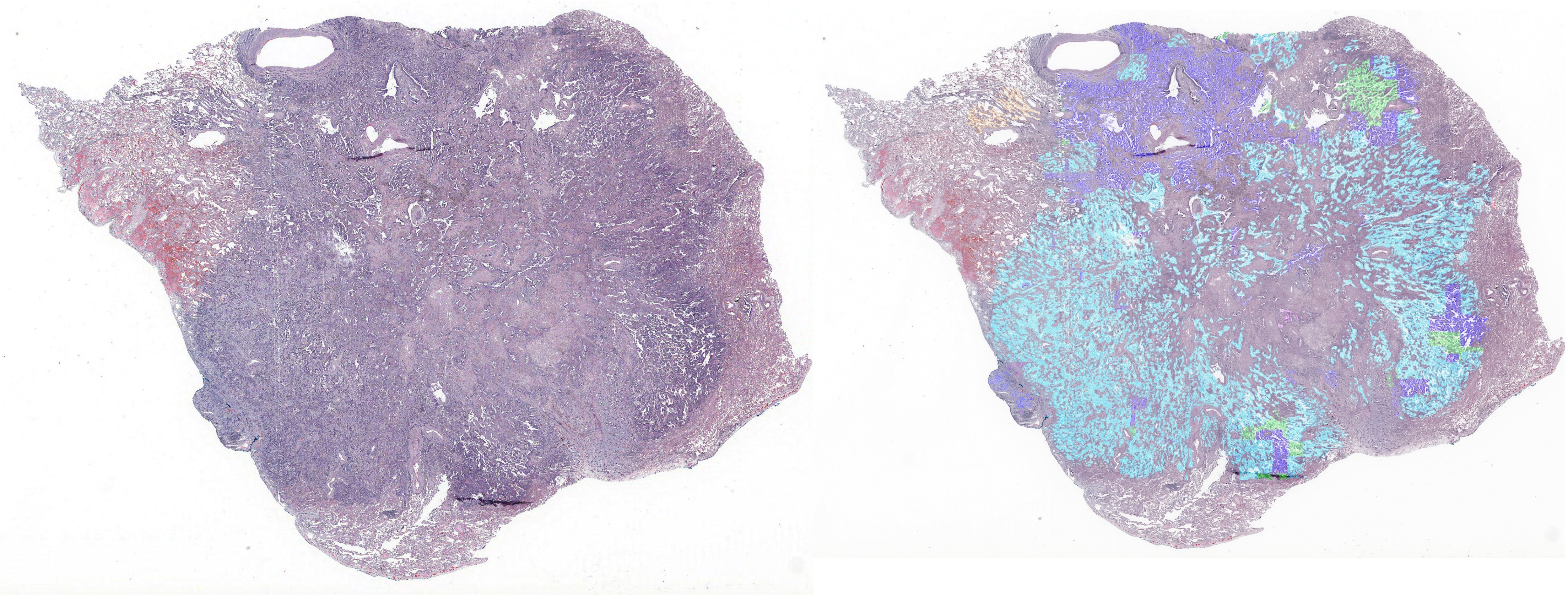

%						
AI	22	2	69	6	0	1

ACINAR
PAPILLARY
SOLID
LEPIDIC
MICROPAPILLARY
COMPLEX GLAND

Figure S9. Further example of whole-slide image inference using PATQUANT.

Metrics are provided. Left side: original H&E image. Right side: overlay of PATQUANT pattern segmentation map on the original H&E image.

Created in BioRender. Tolkach, I. (2025) https://BioRender.com/q10s060

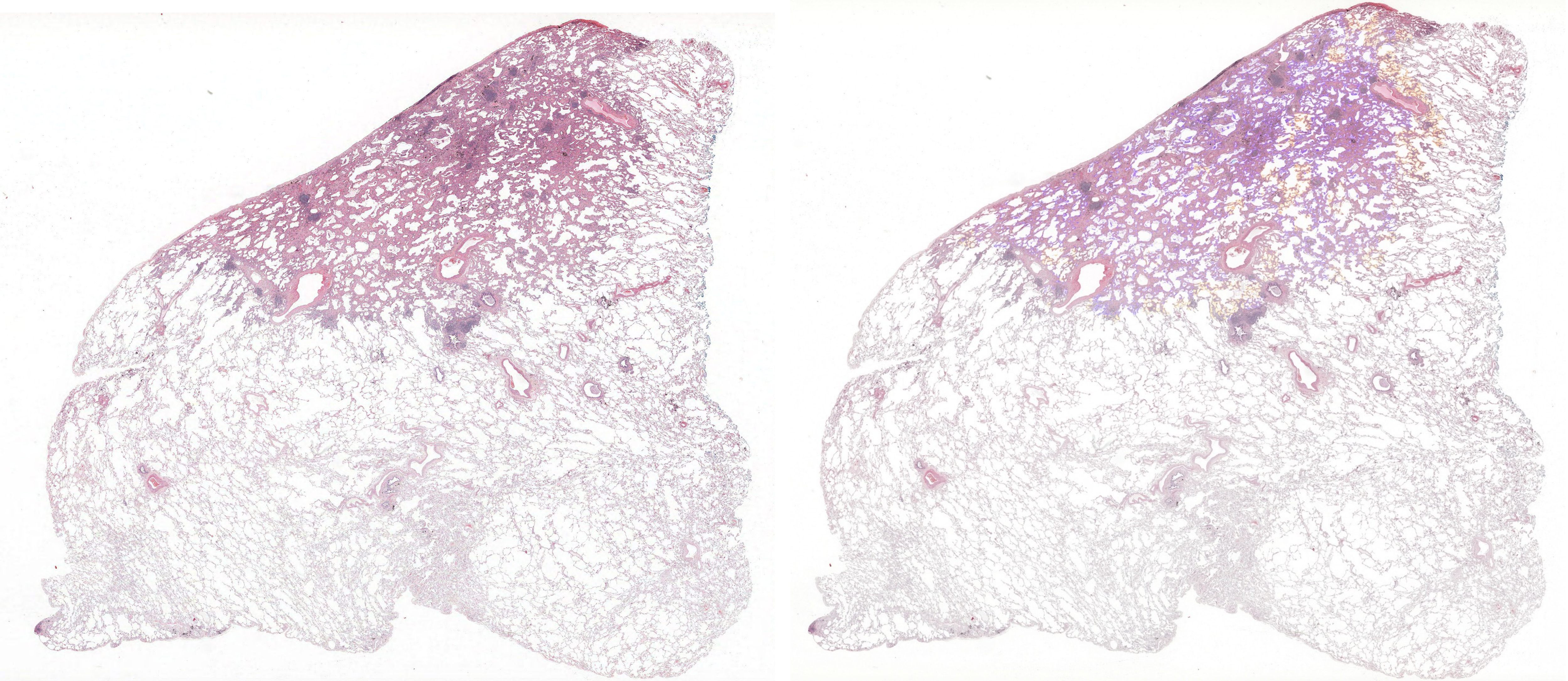

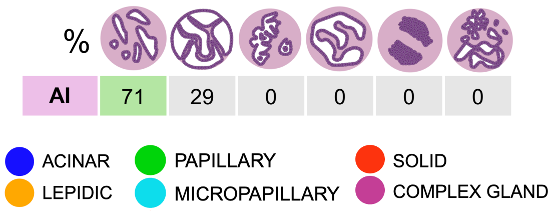

Figure S10. Further example of whole-slide image inference using PATQUANT.

Metrics are provided. Left side: original H&E image. Right side: overlay of PATQUANT pattern segmentation map on the original H&E image.

Created in BioRender. Tolkach, I. (2025) https://BioRender.com/q10s060

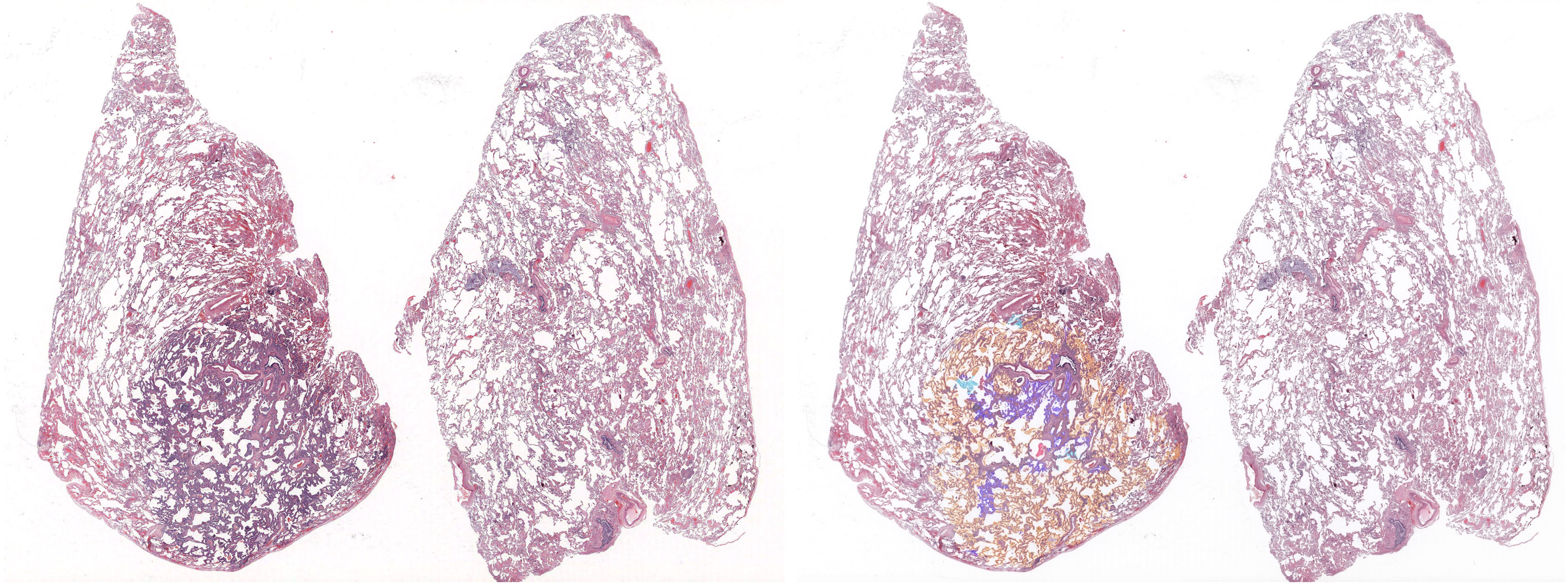

%						
AI	19	78	3	0	0	0

ACINAR
LEPIDIC
PAPILLARY
MICROPAPILLARY
SOLID
COMPLEX GLAND

Figure S11. Further example of whole-slide image inference using PATQUANT.

Metrics are provided. Left side: original H&E image. Right side: overlay of PATQUANT pattern segmentation map on the original H&E image.

Created in BioRender. Tolkach, I. (2025) https://BioRender.com/q10s060

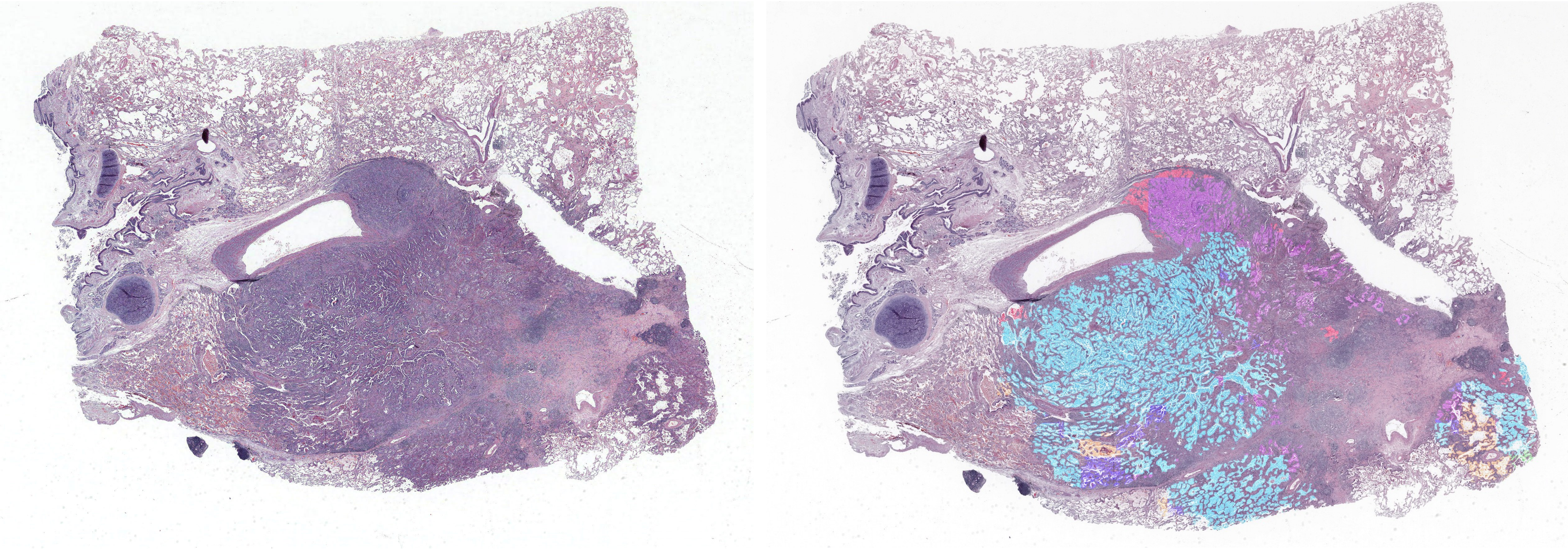

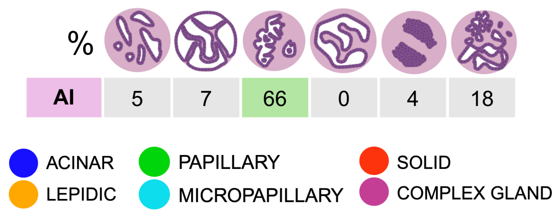

Figure S12. Further example of whole-slide image inference using PATQUANT.

Metrics are provided. Left side: original H&E image. Right side: overlay of PATQUANT pattern segmentation map on the original H&E image.

Created in BioRender. Tolkach, I. (2025) https://BioRender.com/q10s060

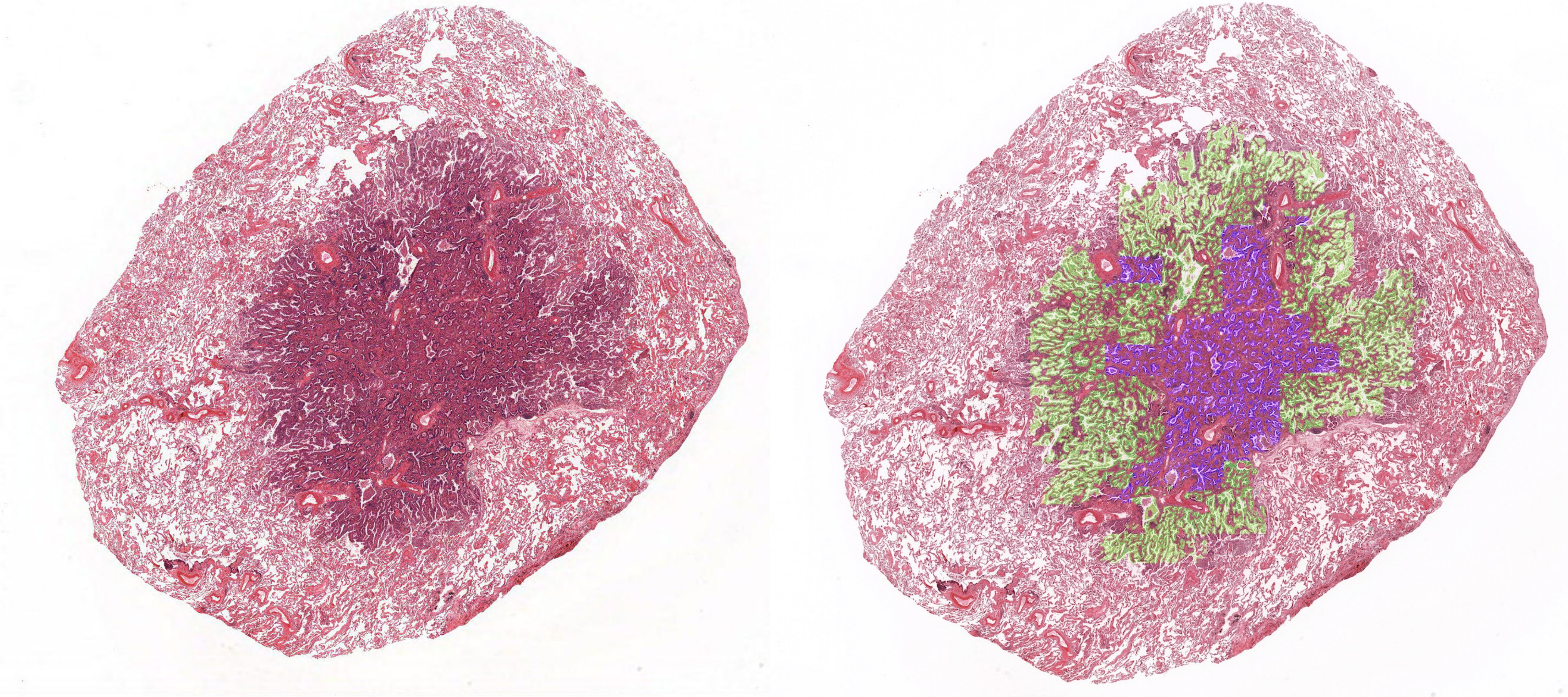

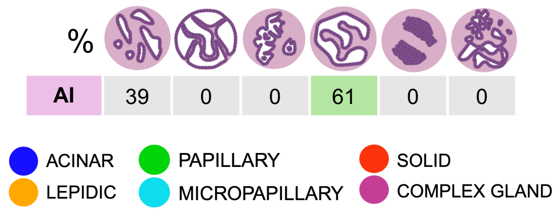

Figure S13. Further example of whole-slide image inference using PATQUANT.

Metrics are provided. Left side: original H&E image. Right side: overlay of PATQUANT pattern segmentation map on the original H&E image.

Created in BioRender. Tolkach, I. (2025) https://BioRender.com/q10s060

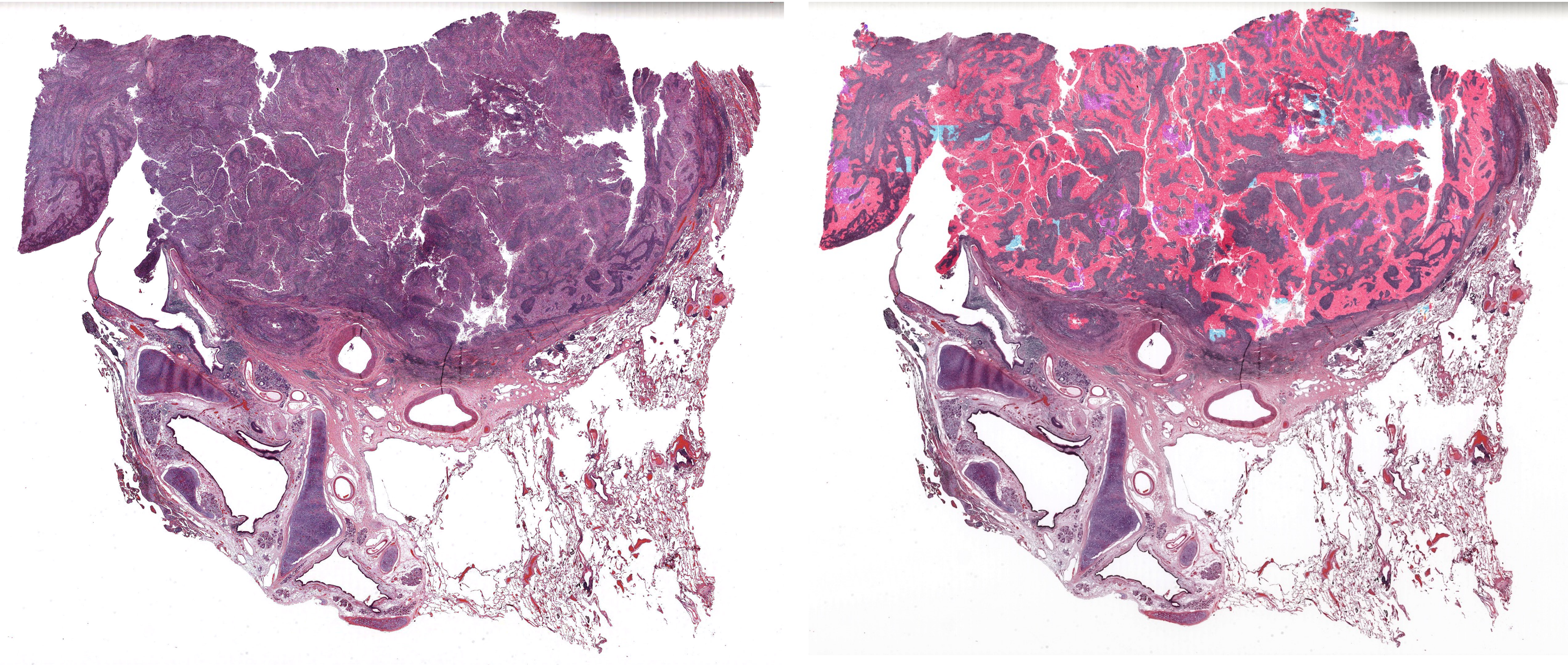

%						
AI	0	0	1	0	94	5

ACINAR
LEPIDIC
PAPILLARY
MICROPAPILLARY
SOLID
COMPLEX GLAND

Figure S14. Further example of whole-slide image inference using PATQUANT.

Metrics are provided. Left side: original H&E image. Right side: overlay of PATQUANT pattern segmentation map on the original H&E image.

Created in BioRender. Tolkach, I. (2025) https://BioRender.com/q10s060

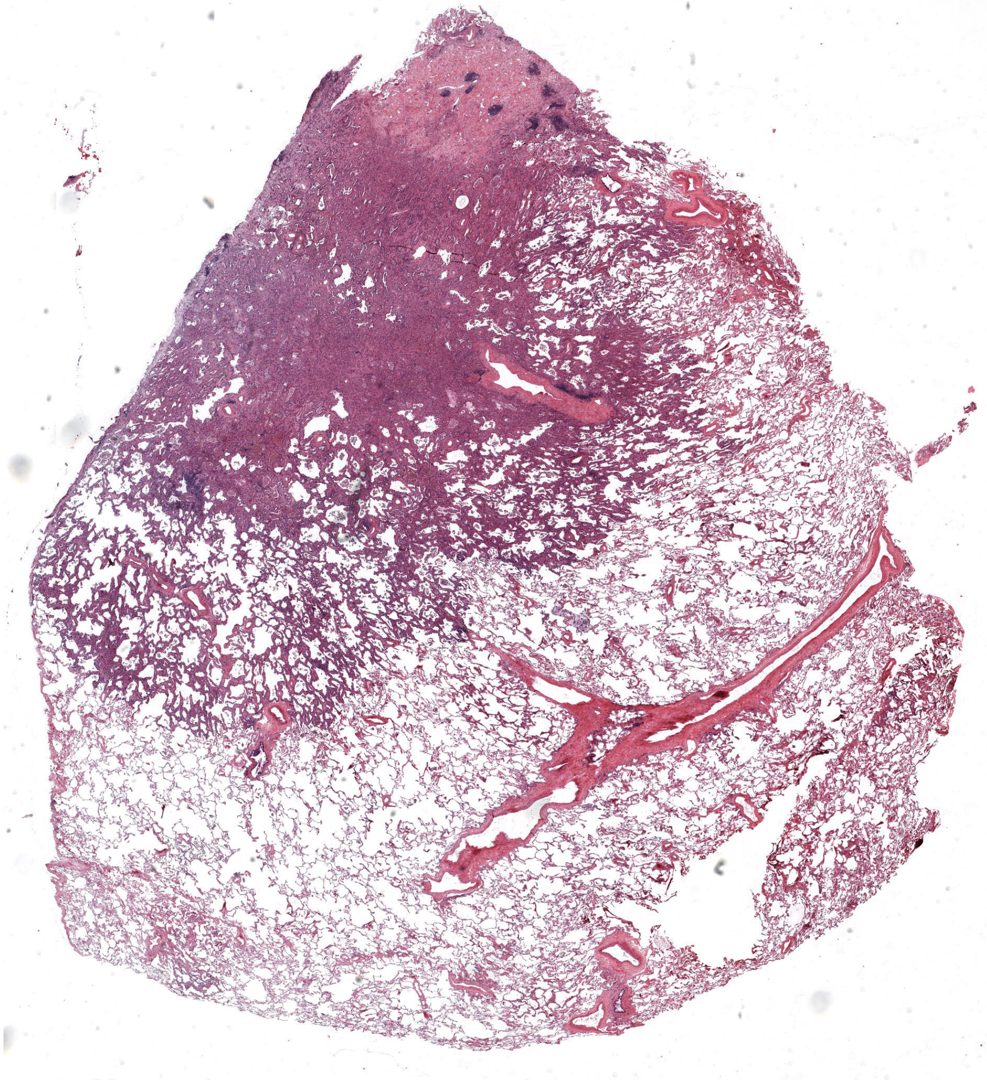

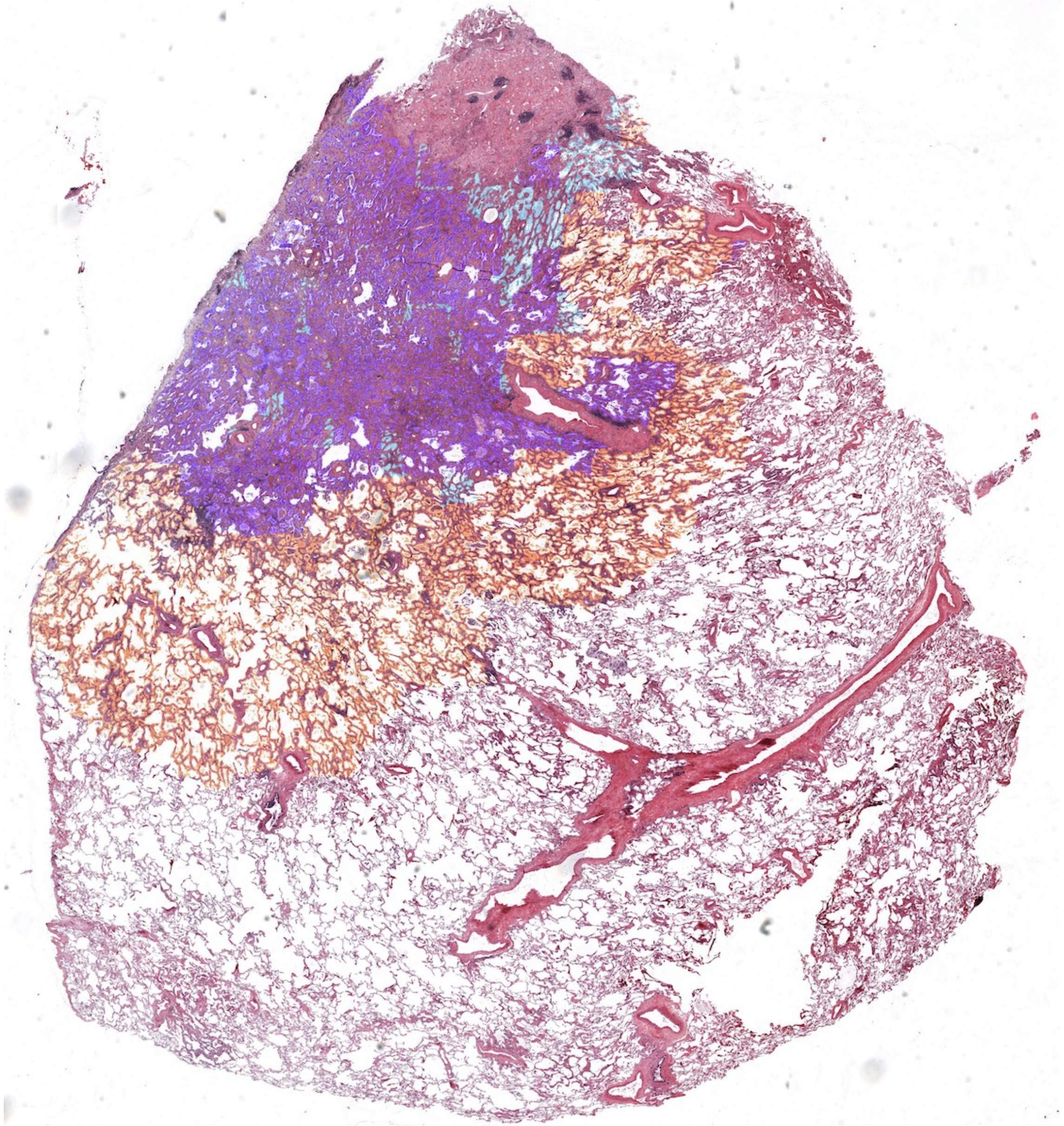

%						
AI	37	54	9	0	0	0

ACINAR PAPILLARY SOLID
LEPIDIC MICROPAPILLARY COMPLEX GLAND

Figure S15. Further example of whole-slide image inference using PATQUANT.

Metrics are provided. Left side: original H&E image. Right side: overlay of PATQUANT pattern segmentation map on the original H&E image.

Created in BioRender. Tolkach, I. (2025) https://BioRender.com/q10s060

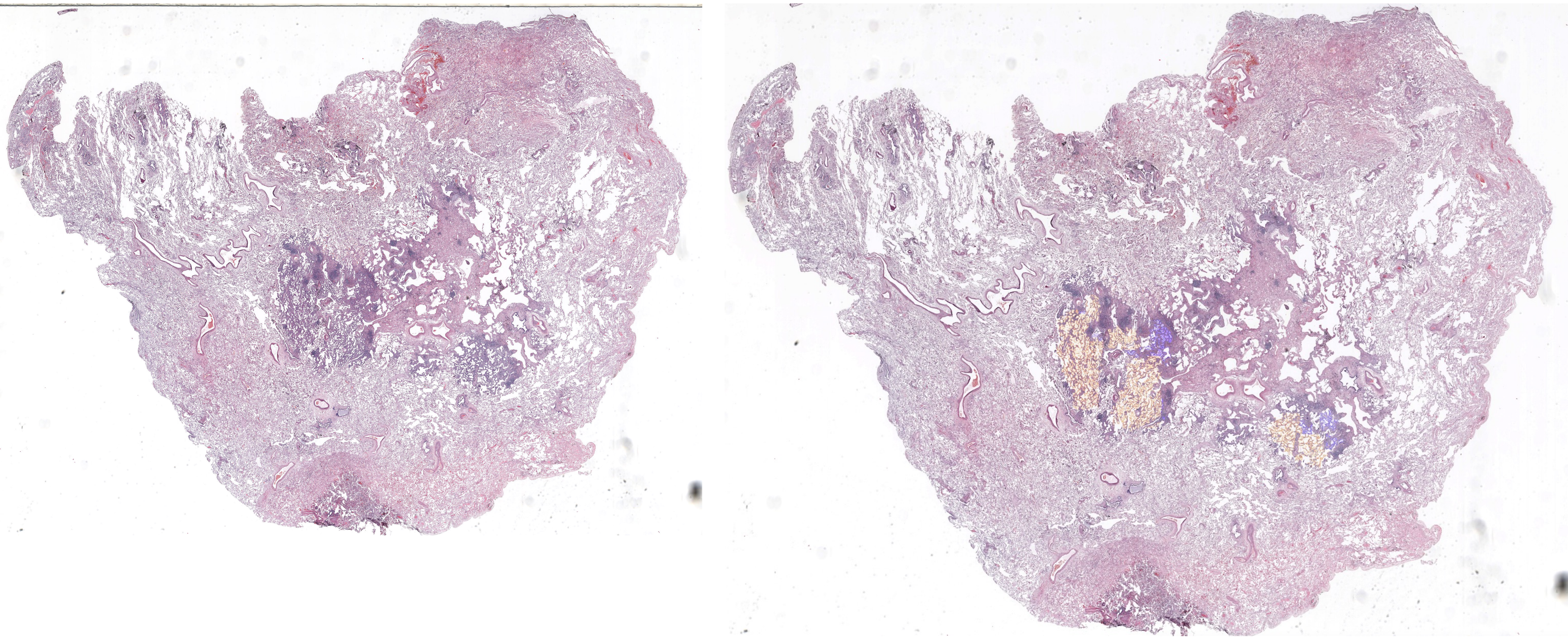

%						
AI	17	83	0	0	0	0

ACINAR, PAPILLARY, SOLID, LEPIDIC, MICROPAPILLARY, COMPLEX GLAND

Figure S16. Further example of whole-slide image inference using PATQUANT.

Metrics are provided. Left side: original H&E image. Right side: overlay of PATQUANT pattern segmentation map on the original H&E image.

Created in BioRender. Tolkach, I. (2025) https://BioRender.com/q10s060

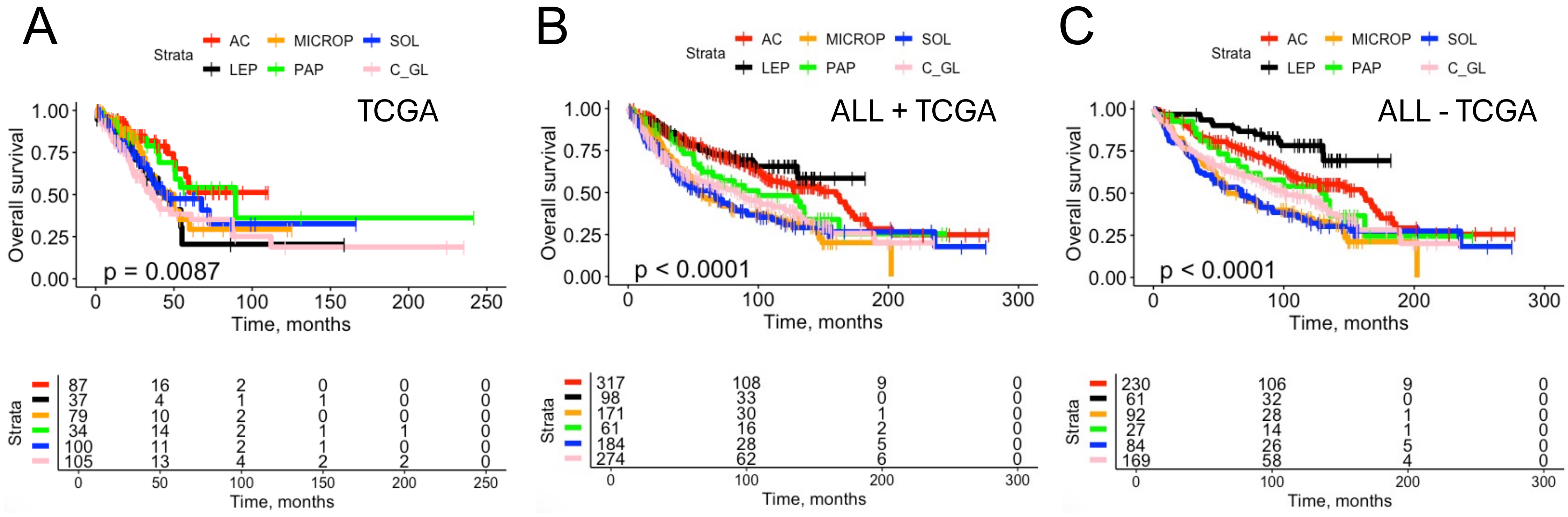

Figure S17. Prognostic study: detailed investigation of TCGA cohort. A. Kaplan-Meier estimates with log-rank test. Cohort: TCGA. Stratification: dominant pattern at case level. Endpoint: overall survival (OS). Note a high-risk prognostic profile of lepidic-predominant tumors, not evident in other cohorts which is attributed to selection biases of TCGA cohort (for more details see Results). **B.** Kaplan-Meier estimates with log-rank test. Cohort: all five cohorts merged (PLCO, NLST, TCGA, UKK, KAMEDA). Stratification: dominant pattern at case level. Endpoint: overall survival (OS). **C.** Kaplan-Meier estimates with log-rank test. Cohort: four cohorts merged (PLCO, NLST, UKK, KAMEDA) excluding TCGA. Stratification: dominant pattern at case level. Endpoint: overall survival (OS). In C a clear low risk prognostic trend of lepidic predominant tumors can be seen, when TCGA cohort excluded. This does not apply to other patterns.

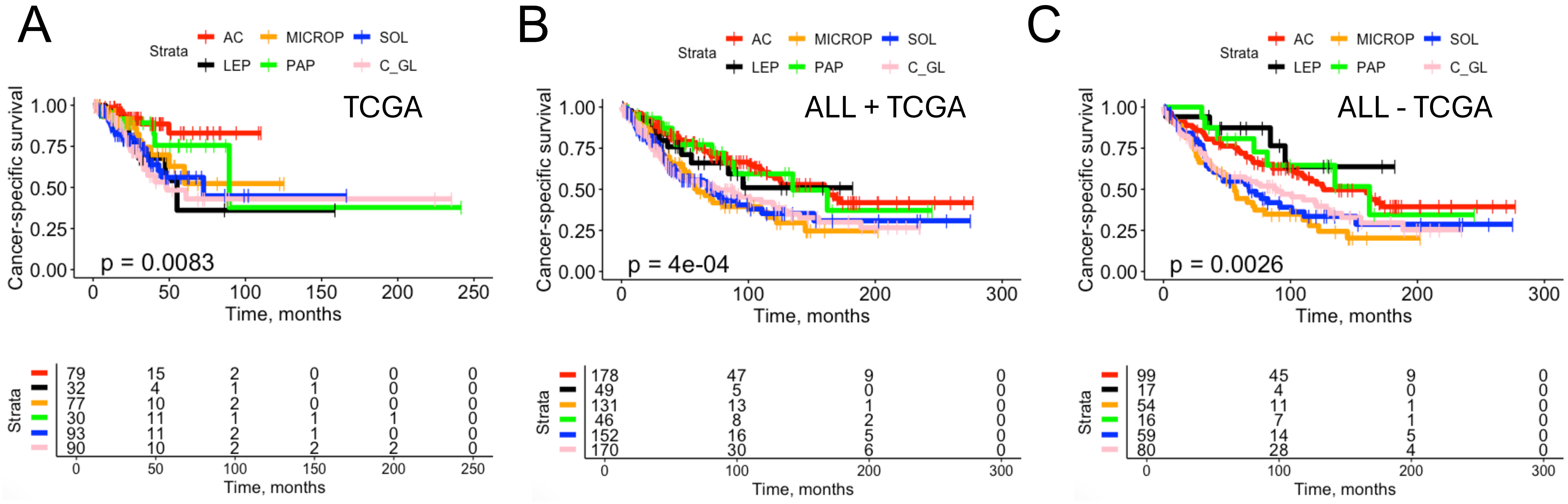

Figure S18. Prognostic study: detailed investigation of TCGA cohort. A. Kaplan-Meier estimates with log-rank test. Cohort: TCGA. Stratification: dominant pattern at case level. Endpoint: cancer-specific survival (CSS). Note a high-risk prognostic profile of lepidic-predominant tumors, not evident in other cohorts which is attributed to selection biases of TCGA cohort (for more details see Results). **B.** Kaplan-Meier estimates with log-rank test. Cohort: all five cohorts merged (PLCO, NLST, TCGA, UKK, KAMEDA). Stratification: dominant pattern at case level. Endpoint: cancer-specific survival (CSS). **C.** Kaplan-Meier estimates with log-rank test. Cohort: four cohorts merged (PLCO, NLST, UKK, KAMEDA) excluding TCGA. Stratification: dominant pattern at case level. Endpoint: cancer-specific survival (OS). In C a clear low risk prognostic trend of lepidic predominant tumors can be seen, when TCGA cohort excluded. This observation does not apply to other patterns.

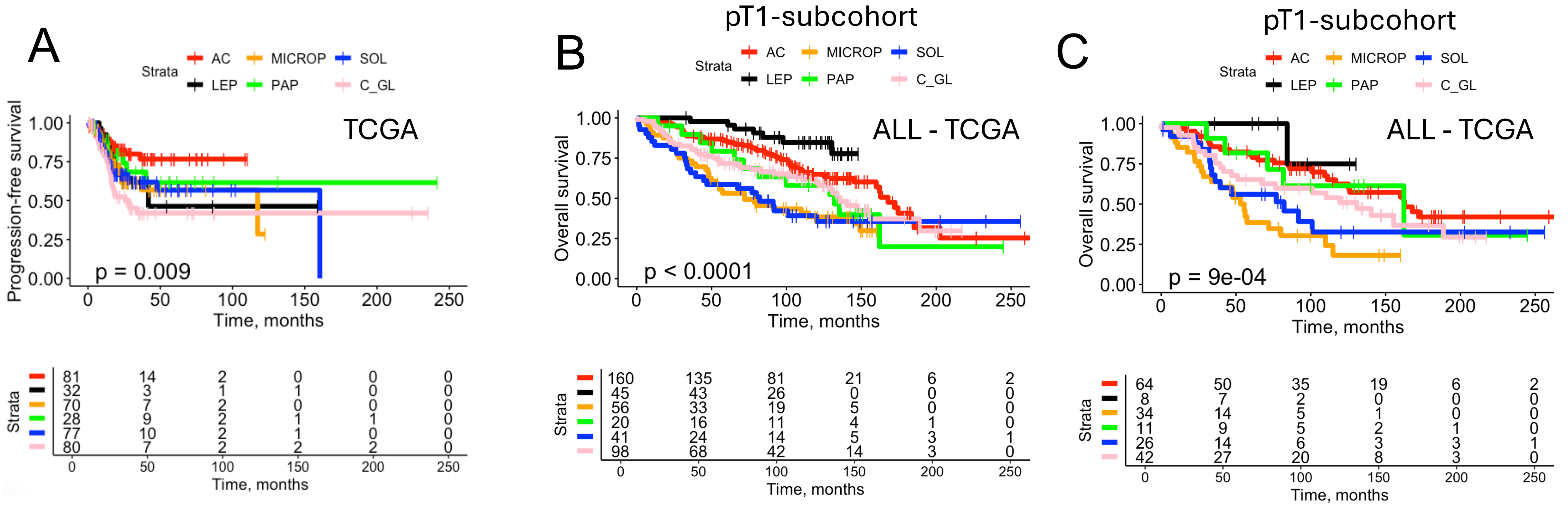

Figure S19. Prognostic study. A. Detailed investigation of TCGA cohort. Kaplan-Meier estimates with log-rank test. Cohort: TCGA. Stratification: dominant pattern at case level. Endpoint: progression-free survival (PFS). Note a high-risk prognostic profile of lepidic-predominant tumors, not evident in other cohorts which is attributed to selection biases of TCGA cohort (for more details see Results). **B.** Kaplan-Meier estimates with log-rank test. Cohort: pT1-subcohort; four cohorts merged excluding TCGA (PLCO, NLST, UKK, KAMEDA);. Stratification: dominant pattern at case level. Endpoint: overall survival (OS). **C.** Kaplan-Meier estimates with log-rank test. Cohort: pT1-subcohort; four cohorts merged excluding TCGA (PLCO, NLST, UKK, KAMEDA). Stratification: dominant pattern at case level. Endpoint: cancer-specific survival (CSS).

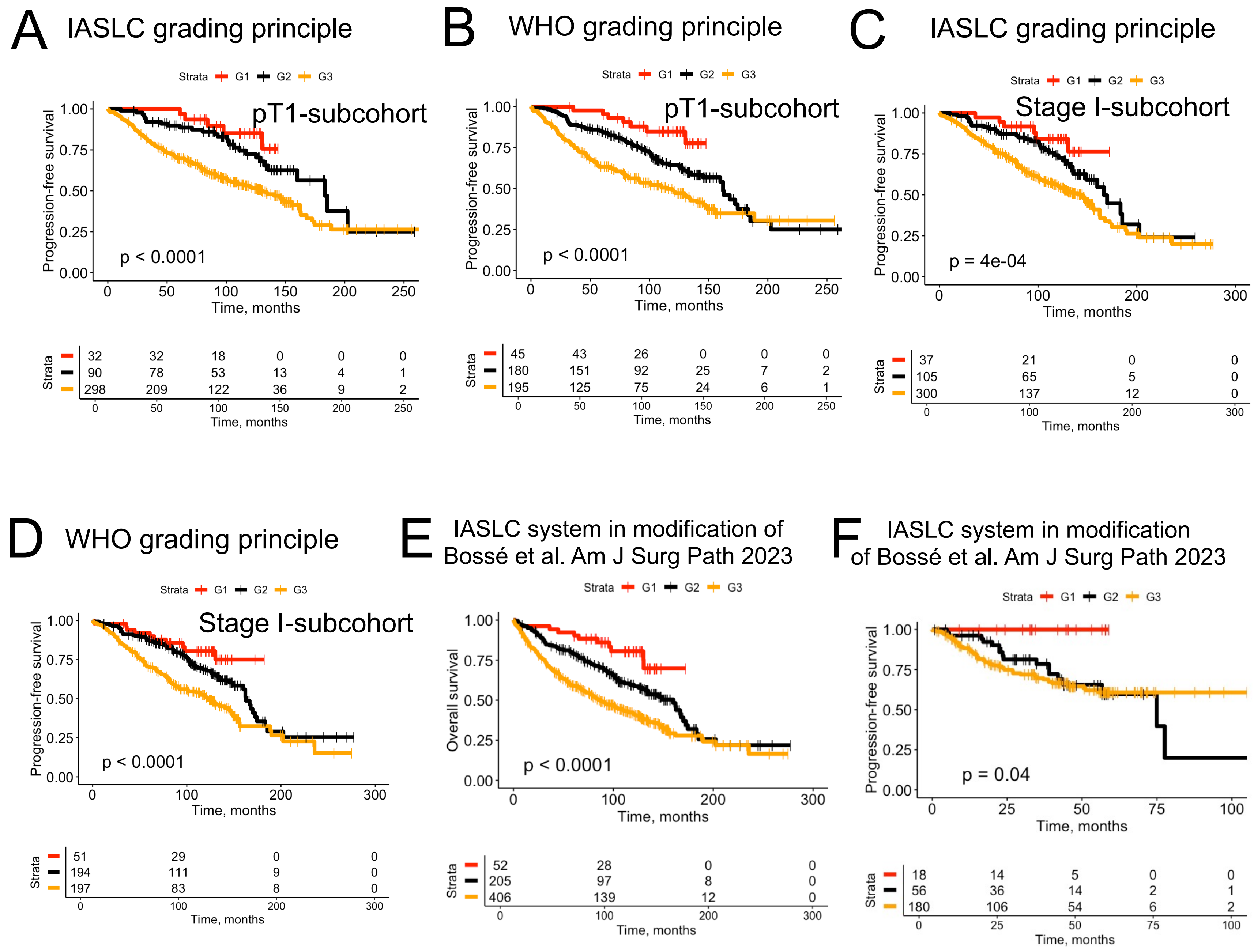

Figure S20. Prognostic study: WHO/IASLC systems for pT1-subcohort and IASLC-proposed system for grading in modification of Bossé et al.

A. Grading according IASLC-proposed grading system considering re-classification of G1 and G2 cases with >20% high-risk patterns (micropapillary, complex glandular, solid) as G3. Kaplan-Meier estimates with log-rank test. Cohort: pT1-subcohort, merged cohort (PLCO, NLST, UKK, KAMEDA). Endpoint: overall survival (OS). **B.** Grading according to WHO-recommended grading system, considering only dominant pattern (G1 – lepidic, G2 – acinar, papillary, G3 – solid, micropapillary, complex glandular). Kaplan-Meier estimates with log-rank test. Cohort: pT1-subcohort, merged cohort (PLCO, NLST, UKK, KAMEDA). Endpoint: overall survival (OS). **C.** Grading according IASLC-proposed grading system considering re-classification of G1 and G2 cases with >20% high-risk patterns (micropapillary, complex glandular, solid) as G3. Kaplan-Meier estimates with log-rank test. Cohort: Stage I subcohort, merged cohort (PLCO, NLST, UKK, KAMEDA). Endpoint: overall survival (OS). Univariate/multivariate Cox analysis results in Table S8. **D.** Grading according to WHO-recommended grading system, considering only dominant pattern (G1 – lepidic, G2 – acinar, papillary, G3 – solid, micropapillary, complex glandular). Kaplan-Meier estimates with log-rank test. Cohort: Stage I subcohort, merged cohort (PLCO, NLST, UKK, KAMEDA). Endpoint: overall survival (OS). Univariate/multivariate Cox analysis results in Table S9. **E.** Kaplan-Meier estimates with log-rank test. Cohort: all four cohorts merged, excluding TCGA (PLCO, NLST, UKK, KAMEDA). Stratification: simplified IASLC-grading (a simplification of IASLC system through removal of the complex glandular pattern from a sum of high-grade patterns when deciding about G1 and G2 grades). Endpoint: overall survival (OS). **F.** Kaplan-Meier estimates with log-rank test. Cohort: all four cohorts merged, excluding TCGA (PLCO, NLST, UKK, KAMEDA). Stratification: simplified IASLC-grading. Endpoint: progression-free survival (OS).

Overall survival

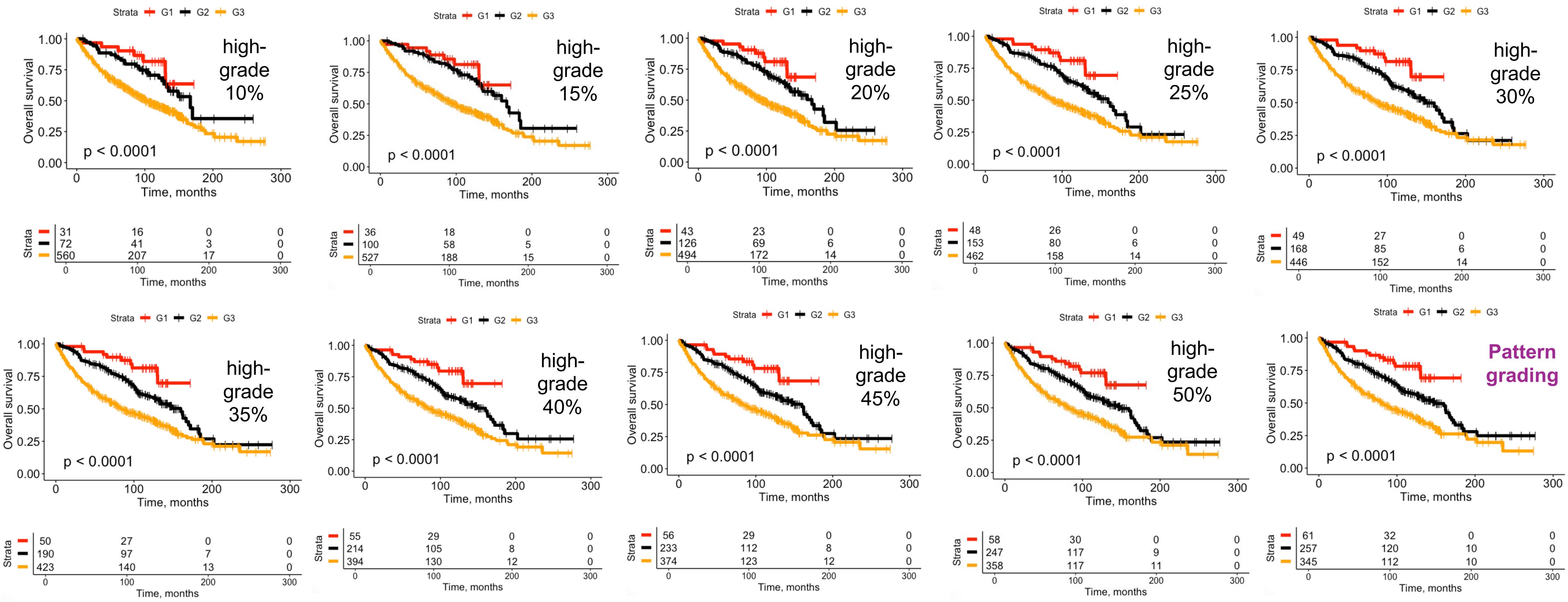

Figure S21. Detecting the optimal high-grade pattern threshold for IASLC grading system (overall survival as endpoint, OS). Cohort: all four cohorts merged, excluding TCGA (PLCO, NLST, UKK, KAMEDA). Micropapillary, complex glandular, and solid patterns were considered as high-grade. The range between 10% and 50% is tested. The result for purely dominant pattern-based grading is provided.

Progression-free survival

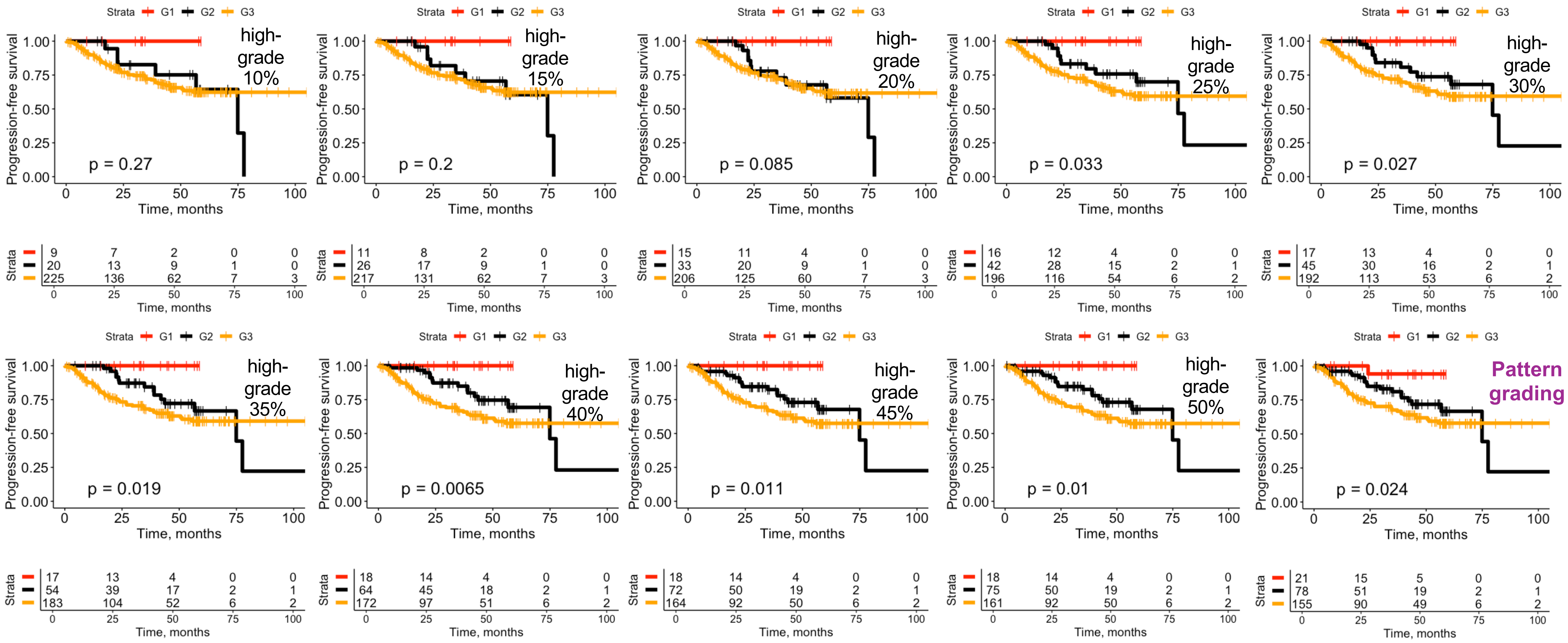

Figure S22. Detecting the optimal high-grade pattern threshold for IASLC grading system (progression-free survival as endpoint, PFS). Cohort: all four cohorts merged, excluding TCGA (PLCO, NLST, UKK, KAMEDA). Micropapillary, complex glandular, and solid patterns were considered as high-grade. The range between 10% and 50% is tested. The result for purely dominant pattern-based grading is provided.

Cancer-specific survival

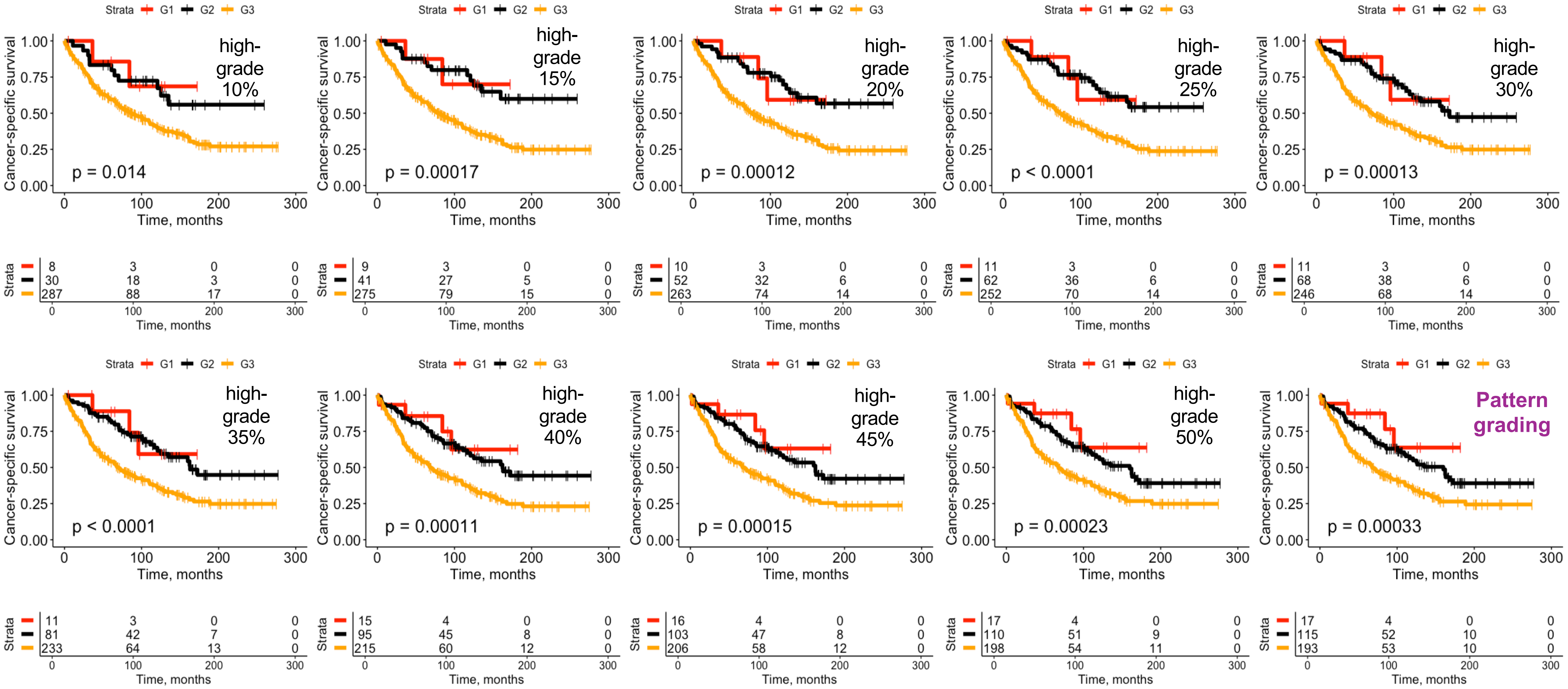

Figure S23. Detecting the optimal high-grade pattern threshold for IASLC grading system (cancer-specific survival as endpoint, CSS). Cohort: all four cohorts merged, excluding TCGA (PLCO, NLST, UKK, KAMEDA). Micropapillary, complex glandular, and solid patterns were considered as high-grade. The range between 10% and 50% is tested. The result for purely dominant pattern-based grading is provided.

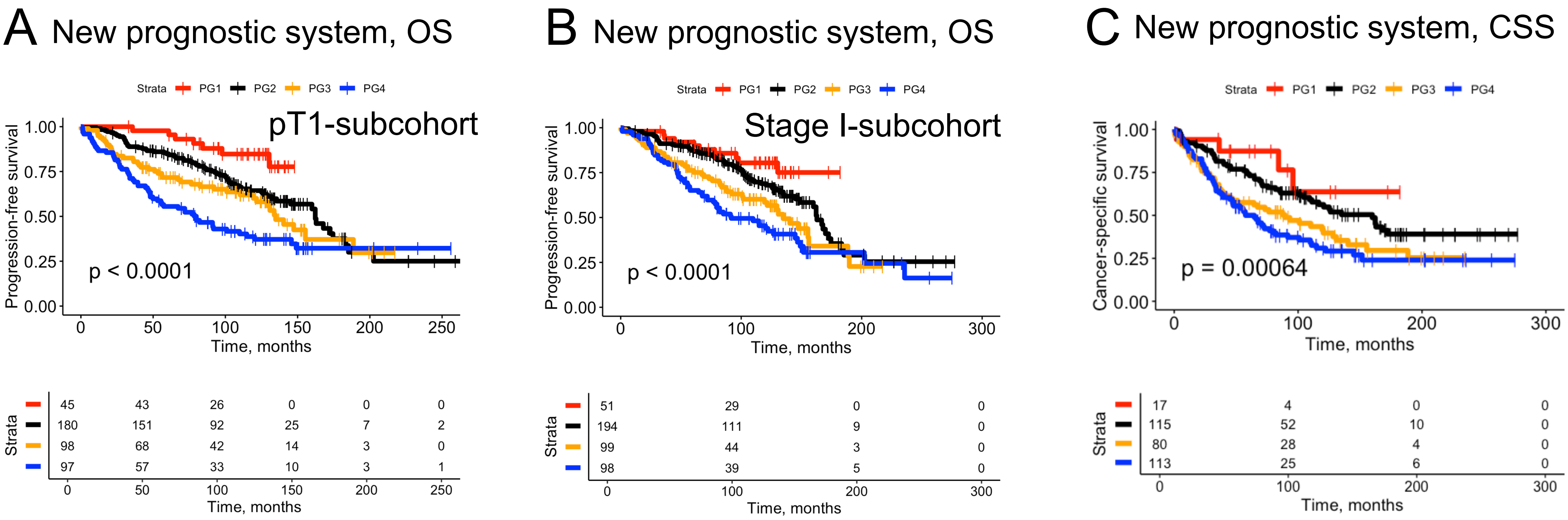

Figure S24. Prognostic study: new four-tier grading system including complex-glandular pattern as a separate grading class.

A. New four-tier grading system. Cohort: pT1-subcohort; merged cohort (PLCO, NLST, UKK, KAMEDA). Endpoint: overall survival (OS). **B.** New four-tier grading system. Cohort: pT1-subcohort; merged cohort (PLCO, NLST, UKK, KAMEDA). Endpoint: overall survival (OS). Univariate/multivariate Cox analysis results in Table S10. **C.** Kaplan-Meier estimates with log-rank test. Cohort: all four cohorts merged, excluding TCGA (PLCO, NLST, UKK, KAMEDA). Stratification: new four-tier grading system (details in Figure 7A). Endpoint: cancer-specific survival (CSS).

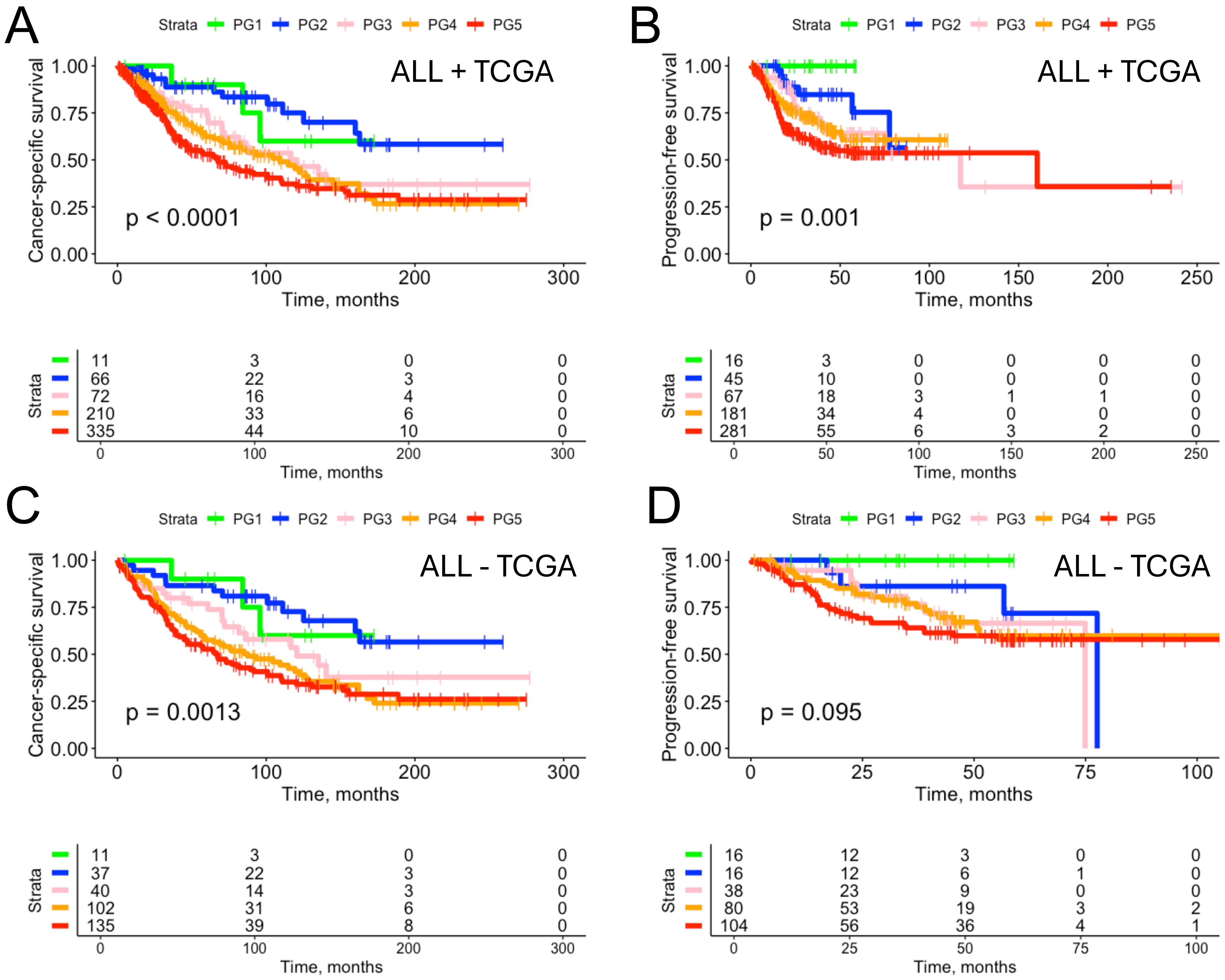

Figure S25. Prognostic study: new prognostic scoring system with five prognostic groups. Details to system are provided in Figure 7D. **A.** Cohort: merged cohort including TCGA (TCGA, PLCO, NLST, UKK, KAMEDA). Endpoint: cancer- specific survival (CSS). **B.** Cohort: merged cohort including TCGA (TCGA, PLCO, NLST, UKK, KAMEDA). Endpoint: progression-free survival (PFS). **C.** Cohort: merged cohort excluding TCGA (PLCO, NLST, UKK, KAMEDA). Endpoint: cancer-specific survival (CSS). **D.** Cohort: merged cohort excluding TCGA (PLCO, NLST, UKK, KAMEDA). Endpoint: progression-free survival (PFS). Results with overall survival as endpoint are provided in Figure 7E,F.

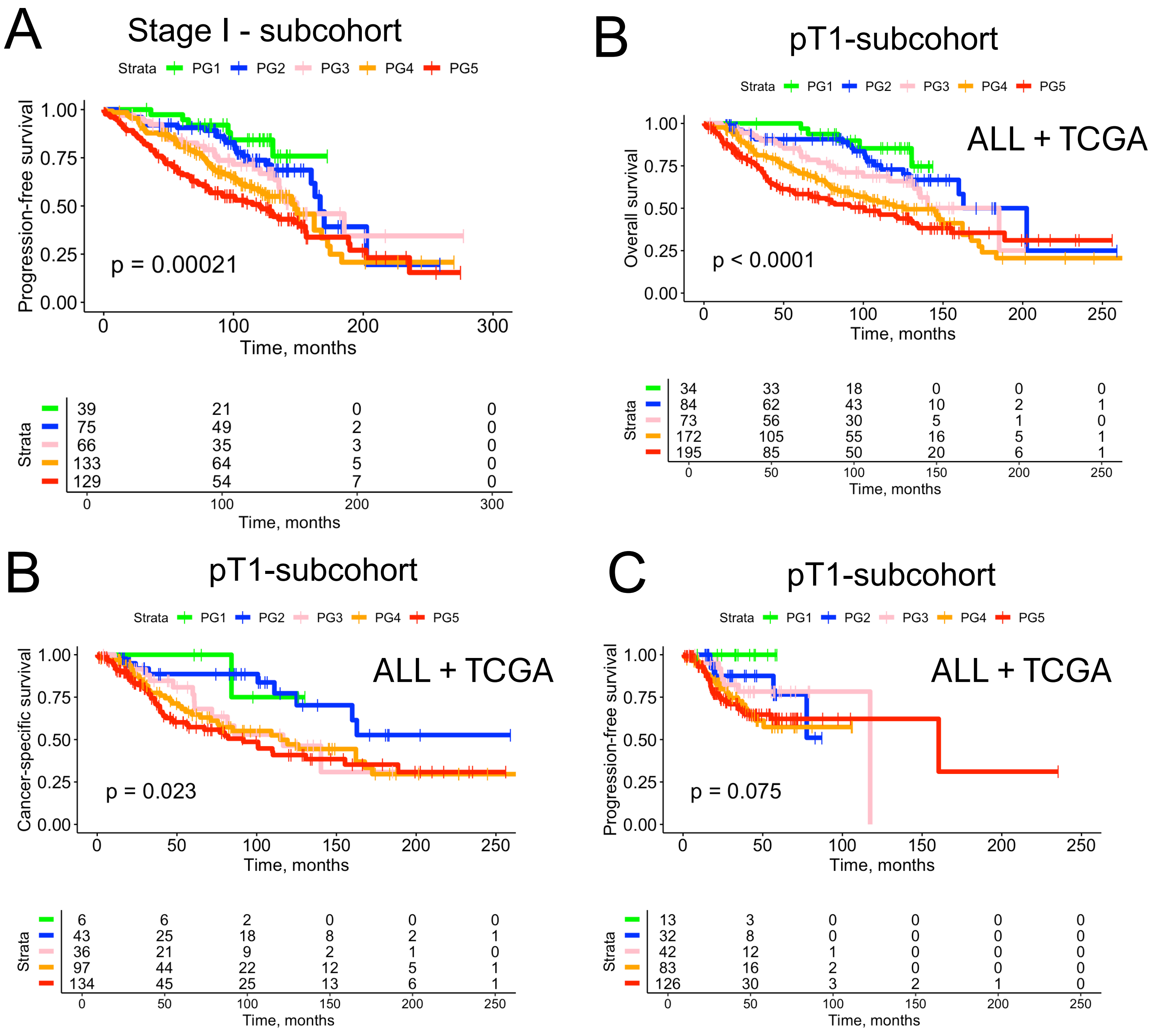

Figure S26. Prognostic study: new prognostic scoring system with five prognostic groups (subcohorts of patients with Stage I and pT1). Details to system are provided in Figure 7D. **A.** Cohort: Stage I-subcohort, merged cohort excluding TCGA (PLCO, NLST, UKK, KAMEDA). Endpoint: overall survival (OS). Univariate/multivariate Cox analysis results in Table S11. **B.** Cohort: pT1-subcohort, merged cohort including TCGA (TCGA, PLCO, NLST, UKK, KAMEDA). Endpoint: overall survival (OS). **C.** Cohort: pT1-subcohort, merged cohort including TCGA (TCGA, PLCO, NLST, UKK, KAMEDA). Endpoint: cancer-specific survival (CSS). **D.** Cohort: pT1-subcohort, merged cohort including TCGA (TCGA, PLCO, NLST, UKK, KAMEDA). Endpoint: progression-free survival (PFS).

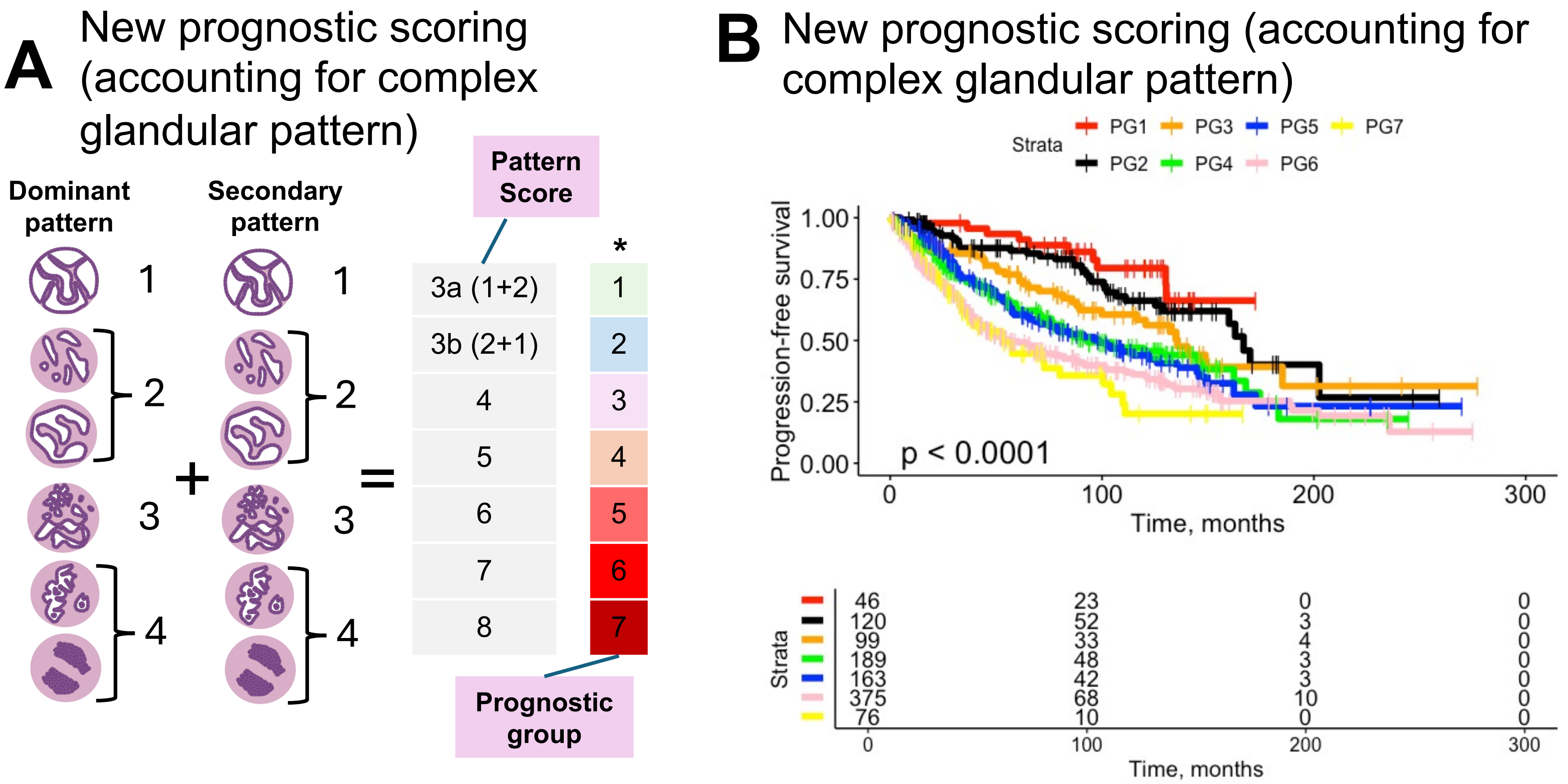

Figure S27. Prognostic study: investigation of the extended version of the new prognostic scoring system. A. New prognostic scoring system is proposed (see also Figure 7D), inspired by Gleason grading in prostate cancer. Score is a sum of single grades for dominant and secondary pattern. Score 3 is subdivided into 3a (1+2) and 3b (2+1). Scoring results in five prognostic groups. The group with score 2 (*) – purely lepidic tumors – is not shown due to low relevance in diagnostic routine but might apply in such cases. This modification accounts for moderate risk profile of complex glandular pattern compared to simpler version (Figure 7D). B. Kaplan Meier estimates with log-rank test. Cohort: merged cohort including TCGA (TCGA, PLCO, NLST, UKK, KAMEDA). Endpoint: overall survival (OS). This modification appears to be too complex and does not seem to bring an additional benefit.

Created in BioRender. Tolkach, I. (2025) https://BioRender.com/q10s060

Table S1. Univariate Cox analysis for Figures 6A-C (dominant pattern, pattern-based stratification)

**Endpoint: OS (663/346)**	*HR*	*95%CI low*	*95%CI high*	*p-level*	
Lepidic	1.0				
Acinar	2.0	1.2	3.4	0.01339	*
Papillary	2.7	1.3	5.4	0.00542	**
Complex glandular	3.1	1.8	5.4	4.29E-05	***
Micropapillary	4.1	2.3	7.2	9.10E-07	***
Solid	3.8	2.2	6.8	3.71E-06	***

**Endpoint: CSS (325/179)**	*HR*	*95%CI low*	*95%CI high*	*p-level*	
Lepidic	1.0				
Acinar	2.2	0.8	6.1	0.1281	
Papillary	1.9	0.5	6.4	0.3227	
Complex glandular	3.4	1.2	9.3	0.0193	*
Micropapillary	4.4	1.6	12.3	0.0052	**
Solid	3.6	1.3	10.2	0.0147	*

**Endpoint: PFS (254/74)**	*HR*	*95%CI low*	*95%CI high*	*p-level*	
Lepidic	1.0				
Acinar	5.9	0.8	44.3	0.0847	.
Papillary	2.9	0.3	32.5	0.3782	
Complex glandular	7.4	1.0	54.2	0.0495	*
Micropapillary	7.3	0.9	56.4	0.0574	.
Solid	10.2	1.3	77.8	0.0249	*

Comment: Endpoint (Xn,Yn): Xn - number patients in analysis, Yn - number of events
Significance codes: 0 ‘***’; 0.001 ‘**’; 0.01 ‘*’; 0.05 ‘.’; 0.1 ‘ ’

Table S2. Univariate and multivariate Cox analysis for Figure 6D (IASLC grading; OS endpoint)

**Endpoint: OS (663/346)**	*HR*	*95%CI low*	*95%CI high*	*p-level*	
G1	1.0				
G2	1.9	0.9	3.8	0.082	.
G3	3.6	1.9	7.0	0.00015	***

**Endpoint: OS (645/336)**	*HR*	*95%CI low*	*95%CI high*	*p-level*	
G1	1.0				
G2	1.7	0.8	3.5	0.183815	
G3	2.7	1.4	5.6	0.005276	**
pT1	1.0				
pT2	1.5	1.2	1.9	0.000548	***
pT3	2.1	1.3	3.3	0.001531	**
pT4	3.6	2.3	5.5	5.59E-09	***
pN0	1.0				
pN1	1.9	1.4	2.6	6.98E-05	***
pN2	2.6	1.9	3.6	1.32E-09	***
AGE	1.03	1.01	1.05	0.001257	**

Comment: Endpoint (Xn,Yn): Xn - number patients in analysis, Yn - number of events
Significance codes: 0 ‘***’; 0.001 ‘**’; 0.01 ‘*’; 0.05 ‘.’; 0.1 ‘ ’

Table S3. Univariate and multivariate Cox analysis for Figure 6E (WHO grading/dominant pattern-based; OS endpoint)

**Endpoint: OS (663/346)**	*HR*	*95%CI low*	*95%CI high*	*p-level*	
G1	1.0				
G2	2.102	1.207	3.661	0.00869	**
G3	3.625	2.109	6.231	3.14E-06	***

**Endpoint: OS (645/336)**	*HR*	*95%CI low*	*95%CI high*	*p-level*	
G1	1.0				
G2	1.9	1.1	3.4	0.027758	*
G3	2.7	1.5	4.7	0.000649	***
pT1	1.0				
pT2	1.5	1.2	1.9	0.001271	**
pT3	1.9	1.2	3.0	0.005119	**
pT4	3.5	2.3	5.4	1.00E-08	***
pN0	1.0				
pN1	1.9	1.4	2.6	8.84E-05	***
pN2	2.6	1.9	3.6	1.21E-09	***
AGE	1.03	1.01	1.05	0.001506	**

Comment: Endpoint (Xn,Yn): Xn - number patients in analysis, Yn - number of events
Significance codes: 0 ‘***’; 0.001 ‘**’; 0.01 ‘*’; 0.05 ‘.’; 0.1 ‘ ’

Table S4. Univariate and multivariate Cox analysis for Suppl. Figure 19 (IASLC grading in Bossé et al. modification; OS endpoint)

**Endpoint: OS (663/346)**	*HR*	*95%CI low*	*95%CI high*	*p-level*	
G1	1.0				
G2	2.2	1.2	4.2	0.0122	*
G3	3.7	2.0	6.8	2.16E-05	***

**Endpoint: OS (645/336)**	*HR*	*95%CI low*	*95%CI high*	*p-level*	
G1	1.0				
G2	2.0	1.0	3.8	0.038778	*
G3	2.8	1.5	5.3	1.54E-03	**
pT1	1.0				
pT2	1.5	1.2	1.9	0.000973	***
pT3	2.0	1.2	3.1	0.003711	**
pT4	3.7	2.4	5.6	2.61E-09	***
pN0	1.0				
pN1	1.9	1.4	2.6	0.000113	***
pN2	2.6	1.9	3.5	2.00E-09	***
AGE	1.03	1.01	1.05	0.001026	**

Comment: Endpoint (Xn,Yn): Xn - number patients in analysis, Yn - number of events
Significance codes: 0 ‘***’; 0.001 ‘**’; 0.01 ‘*’; 0.05 ‘.’; 0.1 ‘ ’

Table S5. Univariate and multivariate Cox analysis for Figure 7C (4-tier; OS endpoint)

**Endpoint: OS (663/346)**	*HR*	*95%CI low*	*95%CI high*	*p-level*	
G1	1.0				
G2	2.1	1.2	3.7	0.00865	**
G3	3.2	1.8	5.6	5.73E-05	***
G4	4.1	2.3	7.1	6.46E-07	***

**Endpoint: OS (645/336)**	*HR*	*95%CI low*	*95%CI high*	*p-level*	
G1	1.0				
G2	1.9	1.1	3.4	0.027772	*
G3	2.4	1.3	4.3	3.36E-03	**
G4	3.0	1.7	5.3	2.37E-04	***
pT1	1.0				
pT2	1.5	1.2	1.9	0.001512	**
pT3	1.9	1.2	3.0	0.005275	**
pT4	3.4	2.2	5.2	2.24E-08	***
pN0	1.0				
pN1	1.9	1.4	2.6	0.000123	***
pN2	2.7	1.9	3.6	6.76E-10	***
AGE	1.03	1.01	1.05	0.001341	**

Comment: Endpoint (Xn,Yn): Xn - number patients in analysis, Yn - number of events
Significance codes: 0 ‘***’; 0.001 ‘**’; 0.01 ‘*’; 0.05 ‘.’; 0.1 ‘ ’

Table S6. Univariate and multivariate Cox analysis for Figure 7E
(new prognostic scoring; ALL-TCGA; OS endpoint)

**Endpoint: OS (663/346)**	*HR*	*95%CI low*	*95%CI high*	*p-level*	
PG1	1.0				
PG2	1.7308	0.7836	3.823	0.174862	
PG3	2.3804	1.0942	5.179	0.02875	*
PG4	3.5948	1.7501	7.384	0.000494	***
PG5	4.8553	2.3774	9.916	1.44E-05	***

**Endpoint: OS (645/336)**	*HR*	*95%CI low*	*95%CI high*	*p-level*	
PG1	1.0				
PG2	1.4	0.7	3.0	0.336765	
PG3	1.8	0.9	3.8	0.108484	
PG4	2.5	1.3	4.9	0.009275	**
PG5	2.8	1.4	5.6	2.92E-03	**
pT1	1.0				
pT2	1.5	1.2	1.9	0.001194	**
pT3	2.0	1.3	3.2	0.002334	**
pT4	3.5	2.3	5.3	1.28E-08	***
pN0	1.0				
pN1	1.9	1.4	2.6	8.48E-05	***
pN2	2.5	1.8	3.4	5.84E-09	***
AGE	1.03	1.01	1.05	0.000694	***

Comment: Endpoint (Xn,Yn): Xn - number patients in analysis, Yn - number of events
Significance codes: 0 ‘***’; 0.001 ‘**’; 0.01 ‘*’; 0.05 ‘.’; 0.1 ‘ ’

Table S7. Univariate and multivariate Cox analysis for Figure 7F (new prognostic scoring; ALL+TCGA; OS endpoint)

**Endpoint: OS (1068/487)**	*HR*	*95%CI low*	*95%CI high*	*p-level*	
PG1	1.0				
PG2	1.6	0.8	3.2	0.185333	
PG3	2.4	1.2	4.7	0.011654	*
PG4	3.3	1.7	6.2	0.000303	***
PG5	4.6	2.5	8.7	2.15E-06	***

**Endpoint: OS (645/336)**	*HR*	*95%CI low*	*95%CI high*	*p-level*	
PG1	1.0				
PG2	1.4	0.7	3.0	0.332154	
PG3	1.9	0.9	4.0	0.069583	.
PG4	2.5	1.3	4.9	0.007777	**
PG5	3.2	1.6	6.3	6.69E-04	***
pT1	1.0				
pT2	1.5	1.2	1.8	0.000221	***
pT3	2.4	1.7	3.4	1.41E-06	***
pT4	3.3	2.3	4.8	2.06E-10	***
pN0	1.0				
pN1	1.9	1.5	2.4	2.67E-07	***
pN2	2.4	1.8	3.1	6.74E-11	***
AGE	1.02	1.01	1.04	0.000273	***

Comment: Endpoint (Xn,Yn): Xn - number patients in analysis, Yn - number of events
Significance codes: 0 ‘***’; 0.001 ‘**’; 0.01 ‘*’; 0.05 ‘.’; 0.1 ‘ ’

Table S8. Univariate and multivariate Cox analysis for Suppl. Fig. 19C
(IASLC grading; OS endpoint; Stage I subcohort)

**Endpoint: OS (442/190)**	*HR*	*95%CI low*	*95%CI high*	*p-level*	
G1	1.0				
G2	2.0	0.8	4.6	0.13	
G3	3.3	1.4	7.4	0.00438	**

**Endpoint: OS (442/190)**	*HR*	*95%CI low*	*95%CI high*	*p-level*	
G1	1.0				
G2	1.9	0.8	4.4	0.1587	
G3	3.1	1.4	7.1	0.00651	**
pT1	1.0				
pT2	1.6	1.2	2.1	0.00386	**
AGE	1.03	1.00	1.05	0.0239	*

Comment: Endpoint (Xn,Yn): Xn - number patients in analysis, Yn - number of events
Significance codes: 0 '***'; 0.001 '**'; 0.01 '*'; 0.05 '.'; 0.1 ' '

Table S9. Univariate and multivariate Cox analysis for Suppl. Fig. 19D (WHO grading/dominant pattern-based; OS endpoint; Stage I subcohort)

**Endpoint: OS (442/190)**	*HR*	*95%CI low*	*95%CI high*	*p-level*	
G1	1.0				
G2	1.8	0.9	3.4	0.08959	.
G3	3.1	1.6	5.9	0.00063	***

**Endpoint: OS (442/190)**	*HR*	*95%CI low*	*95%CI high*	*p-level*	
G1	1.0				
G2	1.8	0.3	1.7	0.096062	.
G3	3.0	0.3	3.3	0.000953	***
pT1	1.0				
pT2	1.5	1.1	2.0	0.010271	*
AGE	1.03	1.00	1.05	0.024378	*

Comment: Endpoint (Xn,Yn): Xn - number patients in analysis, Yn - number of events
Significance codes: 0 ‘***’; 0.001 ‘**’; 0.01 ‘*’; 0.05 ‘.’; 0.1 ‘ ’

Table S10. Univariate and multivariate Cox analysis for Suppl. Fig. 23B (new four-tier grading system; OS endpoint; Stage I subcohort)

**Endpoint: OS (442/190)**	*HR*	*95%CI low*	*95%CI high*	*p-level*	
G1	1.0				
G2	1.8	0.9	3.4	0.089207	.
G3	2.7	1.3	5.3	0.004848	**
G4	3.6	1.8	7.0	0.000208	***

**Endpoint: OS (442/190)**	*HR*	*95%CI low*	*95%CI high*	*p-level*	
G1	1.0				
G2	1.8	0.9	3.4	0.095253	.
G3	2.6	1.3	5.2	0.005508	**
G4	3.4	1.7	6.6	0.000397	***
pT1	1.0				
pT2	1.5	1.1	2.0	0.016364	*
AGE	1.03	1.00	1.05	0.021648	*

Comment: Endpoint (Xn,Yn): Xn - number patients in analysis, Yn - number of events
Significance codes: 0 ‘***’; 0.001 ‘**’; 0.01 ‘*’; 0.05 ‘.’; 0.1 ‘ ’

Table S11. Univariate and multivariate Cox analysis for Suppl. Fig. 25A (new five-tier prognostic scoring system; OS endpoint; Stage I subcohort)

**Endpoint: OS (442/190)**	*HR*	*95%CI low*	*95%CI high*	*p-level*	
PG1	1.0				
PG2	1.9	0.8	4.5	0.17755	
PG3	2.4	1.0	5.8	0.05775	.
PG4	3.1	1.3	7.1	0.00874	**
PG5	4.0	1.7	9.3	0.00108	**

**Endpoint: OS (442/190)**	*HR*	*95%CI low*	*95%CI high*	*p-level*	
PG1	1.0				
PG2	1.8	0.7	4.4	0.20664	
PG3	2.1	0.9	5.2	0.10461	
PG4	3.0	1.3	7.0	0.00941	**
PG5	3.8	1.6	8.7	0.00179	**
pT1	1.0				
pT2	1.5	1.1	2.1	0.00883	**
AGE	1.03	1.01	1.05	0.01281	*

Comment: Endpoint (Xn,Yn): Xn - number patients in analysis, Yn - number of events
Significance codes: 0 '***'; 0.001 '**'; 0.01 '*'; 0.05 '.'; 0.1 ' '
